# Abstracts from the First European Meeting for ATTR Amyloidosis for Doctors and Patients: Paris, France. 02-03 November 2017

**DOI:** 10.1186/s13023-017-0710-5

**Published:** 2017-11-02

## Introduction

### David Adams^1^, Philip Hawkins^2^, Hartmut Schmidt^3^

#### ^1^Department of Neurology, NNERF, Bicêtre Hospital, Assistance Publique - Hôpitaux de Paris, Paris-Sud University, Le Kremlin-Bicêtre, France; ^2^National Amyloidosis Centre, Division of Medicine, University College London, London, UK; ^3^University Hospital of Muenster, Muenster, Germany

ATTR amyloidosis comprises a group of rare multisystem diseases including non-hereditary wild type ATTR amyloidosis (also known as senile cardiac amyloidosis, senile systemic amyloidosis), Familial Amyloid Polyneuropathy (FAP) and Familial Amyloid Cardiomyopathy (FAC).

FAP was first described in the 1950s in Portugal presenting as autosomal dominant disease, whereas wild type ATTR amyloidosis was recognized in the 1980s as a non-hereditary form of restrictive cardiomyopathy in older patients.

ATTR amyloidoses are global diseases that are now being identified in most countries. They are progressive and life-threatening, and diagnosis is usually delayed.

**Fig. 1.**
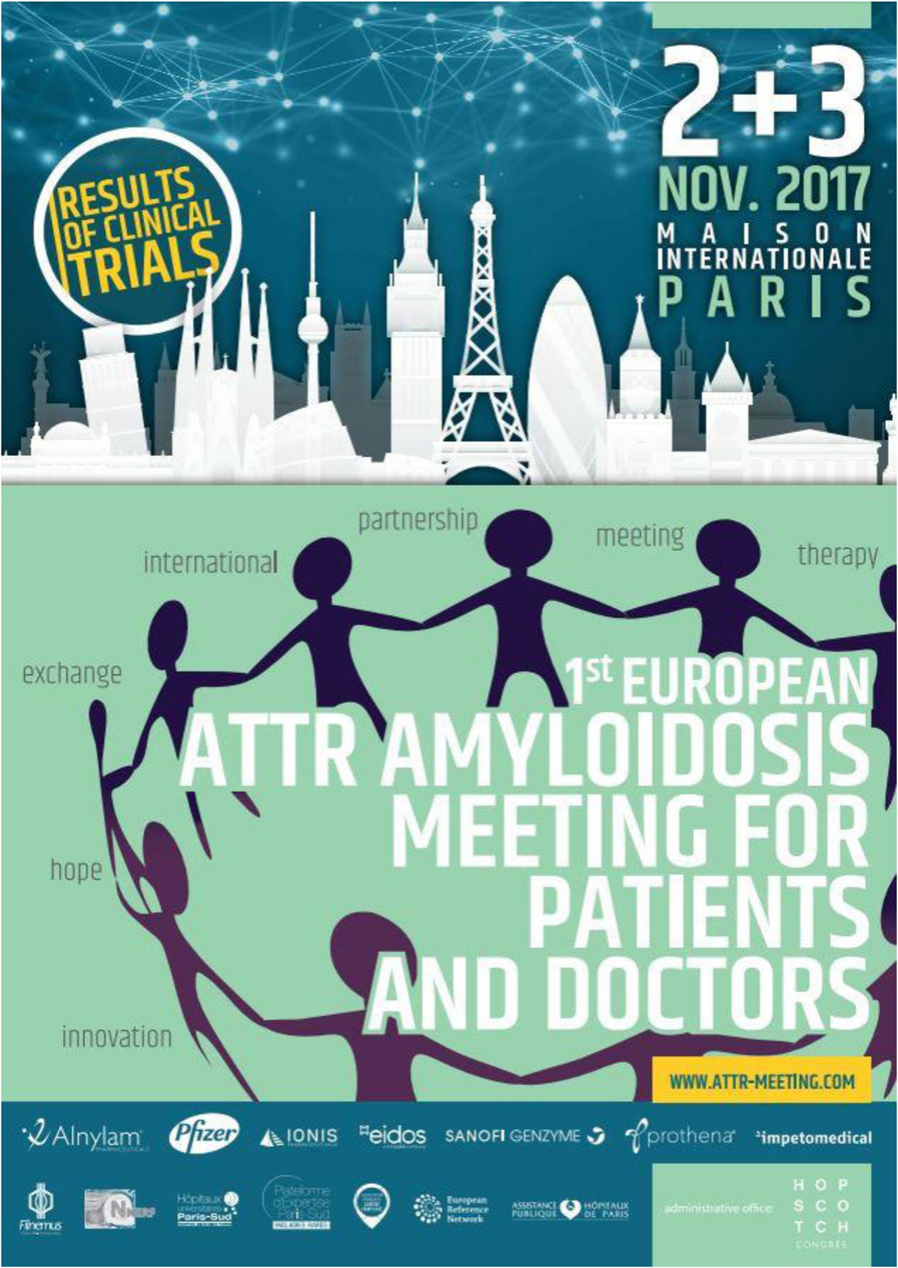
Conference poster

The First European meeting for ATTR amyloidosis for doctors and patients (Fig. 1) will inaugurate a new era in relations between patients, between doctors, and between doctors and patients from many European countries with the ultimate aim of improving diagnosis, treatment and care of this serious disease.

Advocacy and Education experts from international institutions including EURORDIS (the Voice of Rare Disease Patients in Europe), ARC (Amyloidosis Research Centre), and the ISA (International Society of Amyloidosis) will be present.

Since it is the first event of this nature, we propose three interconnected meetings over the course of two days. On Day One, Patients and Doctors will each have their own meeting; on Day Two, Patients and Doctors will share a common session.

During the meeting for specialists, six keynote lectures and 59 abstracts will be presented, comprising 11 oral communications and 48 posters. Final data from two major phase 3 clinical trials for TTR-FAP will be presented.

Eleven national Patients’ Organizations will participate. Attendees will include specialists predominantly from Europe but also from USA, South America and Asia.

## Speaker presentations

### S1 TTR-FAP; where are we now and what are the challenges?

#### Reilly M M (m.reilly@ucl.ac.uk)

##### MRC Centre for Neuromuscular Diseases, UCL Institute of Neurology, Queen Square, London UK

TTR-FAP is a dominantly inherited systemic disease caused by over 100 different pathogenic mutations in the transthyretin (*TTR*) gene. The classic phenotype of TTR-FAP is characterised by a sensory motor neuropathy with varying degrees of autonomic and cardiac involvement. Since the original description of TTR-FAP Met 30 in Portugal, there have been multiple epidemiological and genotype / phenotype studies published. These studies highlight a number of issues including the phenotypic heterogeneity seen even with the same mutation with early onset and late onset cases, the high incidence of certain mutations in individual ethnic groups e.g. TTR Met 30 in Portugal, TTR Ala 60 in Ireland and the UK and the variety of mutations that can be seen in individual countries e.g. UK, France, USA. There is also increasing recognition that TTR amyloidosis is a systemic disease with reports of involvement of almost every organ including the central nervous system, muscle, lungs, kidneys etc. Neuropathy is increasingly described even in mutations originally though just to cause a cardiomyopathy such as TTR Ile 122 and amyloidosis due to wild type TTR.

The development of successful novel therapies has raised a number of important and urgent challenges. These include the importance of early diagnosis particularly in non endemic areas and with the less common TTR mutations and the need to increase awareness of the risk of misdiagnosis especially by neurologists and cardiologists. A further challenge is both the need to define the optimum time to start therapy in an individual patient and the optimum follow up protocol to monitor therapies.

Finally the new genetic therapies will not be cheap and as in many other genetic diseases, a major challenge will be to make these therapies available in a cost effective way internationally.

### S2 Update in epidemiology and phenotypes in TTR-FAP

#### Yesim Parman (parmany@istanbul.edu.tr)

##### Istanbul University, Istanbul Medical Faculty, Neurology Dep., Neuromuscular Unit, Istanbul, Turkey

TTR-FAP is a rare autosomal dominant disorder caused by mutations of the TTR gene with variable penetration. More than 100 different mutations of TTR have been identified worldwide, but the first-described Val30Met mutation remains the most common. The prevalence of different mutations varies according to ethnicity and geographic region. As a rare disease, the European prevalence of amyloidosis (including secondary amyloidosis) was estimated at 47/100000 in 2014. In particular regions of Portugal and Sweden where TTR-FAP is endemic, disease prevalence ranges from 1 in 1000 to 1 in 10 000 people. Smaller endemic foci have also been identified in Cyprus and Majorca. According to data derived from the ATTReuNET questionnaire, Portugal has the highest number of diagnosed, symptomatic cases (~2000) and more than 500 diagnosed asymptomatic carriers of the disease. The gene carrier frequency for Val30Met in the northern parts of Sweden has been recently estimated at 2% . However, the penetrance is low and the onset is late. These facts lead to a lower prevalence of the disease.

Beyond these endemic regions, the incidence of TTR-FAP is much lower in nonendemic regions. France, Italy, and Germany have the most confirmed cases of TTR-FAP. A prevalence of 8.8/1 000 000 has been reported in Sicily. TTR-FAP cases from non-endemic regions are mainly sporadic or scattered as well as genetically and clinically heteregenous.

In endemic regions (Portugal, Cyprus, and Majorca), TTR-FAP often has a younger age of onset, starts before the age 40 with a progressive sensory-motor and autonomic neuropathy, leading to cachexia and eventually death. Frequently, cardiac, renal, and ocular involvement accompany the clinical picture.

In nonendemic areas, the onset is late, after the age of 50 years. A male predominance for the late-onset TTR-FAP has been observed. Although rare, the neuropathy tends to show different clinical chracteristics such as upper limb onset, motor neuropathy, and ataxic features. Typically, sensory and motor neuropathy symptoms are usually associated with mild autonomic symptoms.

TTR-FAP is a rare, yet devastating, systemic disorder with predominant neurologic involvement, with genotypic/phenotypic and epidemiological variability.

References

Parman Y, Adams D, Obici L et al. Sixty years of transthyretin familial amyloid polyneuropathy (TTR-FAP) in Europe: where are we now? A European network approach to defining the epidemiology and management patterns for TTR-FAP. Curr Opin Neurol. 2016 Feb;29 Suppl 1:S3-13.

Adams D. Hereditary and acquired amyloid neuropathies. J Neurol 2001; 248:647 – 657.

Adams D, Lozeron P, Lacroix C. Amyloid neuropathies. Curr Opin Neurol .,2012; 25:564 – 572.

Orphanet Report Series – Rare Disease Collection. Prevalence of rare diseases: bibliographic data. Orphanet. http://www.orpha.net/orphacom/cahiers/docs/GB/Prevalence_of_rare_diseases_by_alphabetical_list.pdf.

Ando Y, Coelho T, Berk JL, et al. Guideline of transthyretin-related hereditary amyloidosis for clinicians. Orphanet J Rare Dis 2013; 8:31.

Suhr OB, Lindqvist P, Olofsson BO, et al. Myocardial hypertrophy and & function are related to age at onset in familial amyloidotic polyneuropathy. Amyloid 2006; 13:154 – 159.

Adams D, Suhr OB, Hund E et al., First European consensus for diagnosis, management, and treatment of transthyretin familial amyloid polyneuropathy. Curr Opin Neurol. 2016 Feb;29 Suppl 1:S14-26.

### S4 Multimodal imaging of cardiac amyloidosis in aTTR

#### Michel Slama (prmslama@gmail.com)

##### NNERF (National Reference Centre for Rare Disease), Paris, France

Cardiac amyloidosis (CA) is the main cause of mortality in many mutation types of aTTR, and therefore is of major prognostic value. CA is initially latent, and remains underdiagnosed and undertreated. Theoretically, the definitive diagnosis should be established by the demonstration of Congo red staining in cardiac biopsy specimens. However, it cannot reasonably be proposed as an early diagnostic tool, considering its invasive character and limited value due to sampling bias , if alternate reliable noninvasive techniques are available, in addition to genetic testing and non-cardiac positive biopsy. This fuelled the search for multimodal imaging (ECG, echocardiography, MRI, scintigraphy) to assess CA.

The diagnosis is easy in the typical forms of CA, with severe Heart Failure with Preserved Ejection fraction (HPEF), microvoltage and/or conduction disorders on the ECG, myocardial thickening and abnormal strain on echocardiography, cardiac uptake of bone scintigraphy tracers, late gadolinium enhancement (LGE) and T1 mapping with extracellular volume fraction measurement with MRI, and markedly elevated cardiac markers (BNP and troponin). This is a situation where cardiac imaging is at its best, each technique does not add much to the other. Bone scintigraphy is the most specific of aTTR CA, and all have a powerful prognostic value. This is also a situation with no validated therapeutic options.

The diagnosis is more difficult for the early stages of CA, where a disease modifying treatment can possibly translate into an improved prognosis. With the recent refinement of MRI, routine detection of discrete global and regional longitudinal strain abnormalities on echocardiography, cardiac uptake of “bone” tracers (99m Tc-DPD, HMDP), or PYP by scintigraphy, it is possible to precisely detect CA. As proposed by Gillmore et al, cardiac ATTR can be defined using a multimodality imaging approach as the combination of symptoms with an echocardiogram consistent with or suggestive of cardiac amyloidosis, grade 2 or 3 cardiac uptake on 99mTc-DPD scintigraphy in the absence of monoclonal gammopathy, or in the presence of monoclonal gammopathy, a cardiac biopsy positive for TTR. This is the situation where multimodal imaging is most useful.

Very early diagnosis, at stages where no morphological changes have yet occurred, is highly desirable, and will probably rely on techniques with very high sensitivity and specificity such as PET and MRI. techniques. Cardiac sympathetic denervation assessed by MIBG scintigraphy occurs more frequently and earlier than myocardial deposits and may be a useful diagnostic tool in early stages of CA.

The prognostic value of clinical and morphological abnormalities (orthostatic hypotension, NYHA functional class > I, QRS duration ≥ 120 ms, polyneuropathy disability score ≥III, interventricular thickness) is now established. (http://www.nnerf.fr/attrtransplantscore) Cardiac sympathetic denervation detected by 123-I-MIBG also has a very powerful additional prognostic value.

Multimodal imaging of cardiac amyloidosis in aTTR has reached a fair level of precision and is used as a daily practice for diagnostic and prognostic assessment, and can be used to follow patients when disease modifying therapies will become available.

### S5 Unmet need for ATTR amyloidosis therapy?

#### Ole B Suhr (ole.Suhr@umu.se)

##### Department of Medicine, Umeå University, Umeå, Sweden

**Current available treatment modalities**

Even though liver transplantation (LTX) since the last 27 years has improved survival for hereditary transthyretin (TTR) amyloid (ATTRm) amyloidosis patients. However subsequent analysis has shown that it is a limited group of patients that is helped by the procedure, foremost early onset ATTR Val30Met amyloidosis patients that is transplanted early after onset of disease. It has also become apparent that ATTR cardiomyopathy and arrhythmia generally are unaffected by the procedure.

Medical treatments with TTR tetramer stabilisers have showed beneficial outcome on neuropathy in two controlled trials. For Tafamidis, the outcome is depending on age at onset of the disease, type of mutation and also on an early start of treatment after disease onset to reach an acceptable outcome. For Diflunisal, the data are more limited, but for both therapies, a progression of the disease are observed in many individuals, and the efficacy on cardiomyopathy has not been proven.

Lately, differences in amyloid fibril composition has been noted, where occurrence of fragmented TTR can be related the phenotype of the ATTR Val30Met mutation and also to the outcome of LTx. It relationship with response to TTR stabilisers has not been evaluated.

**Medical treatments at the horizon**

We hope that the outcome of two controlled clinical trials, both aiming at profoundly suppress TTR synthesis by either interfering RNA or by antisense oligonucleotides, will be available at this meeting. They both profoundly reduce TTR (both mutant and wild type) concentration in the circulation and should thereby halt ATTR formation. From available data from open-label studies, RNA interference appears to be effective for preventing progression of neuropathy irrespective of mutation and patient’s age at disease onset. Both gene-silencing trials evaluate the efficacy of the drugs on neuropathy, thus, ATTR cardiomyopathy are not primarily targeted in the trials.

However, none of the treatments available or under investigation will have an impact on complications caused by local TTRm synthesis within the brain or eye.

**Conclusions**

Substantial progress in the treatment of ATTR amyloidosis has been made since 1990, but many problems remain to be solved before we have met the “Unmet need for ATTR amyloidosis therapy”

### S6 How to monitor TTR-FAP under treatment

#### Giuseppe Vita, Claudia Stancanelli, Anna Mazzeo

##### University of Messina, Messina, Italy

###### **Correspondence:** Giuseppe Vita (vitag@unime.it)

The management of TTR-FAP has expanded considerably in the recent years with the availability of therapeutic options. A multidisciplinary approach is recommended, including the diagnosing physician, a neurologist and a cardiologist at least. Continuing monitoring is crucial and may permit systematic tracing of disease progression.

The use of standardized, reliable, simple and reproducible outcome measures (OMs) provides a common language with which to manage individual patient care and to evaluate the effectiveness of a given treatment. The clinical complexity of TTR-FAP with multisystem involvement does not make it an easy job. Clinical and laboratory assessment should take place no later than 3 months from treatment start, with a full, multidisciplinary consultation at 6 and 12 months. Regular follow-up can help to diminish patient’s anxiety and to detect new manifestations of amyloid deposits.

Different quantitative and reliable OMs have been used in TTR-FAP natural history studies and clinical trials. Gross disease staging includes FAP stage 1 to 3 scale and Polyneuropathy Disability Score. Compound Autonomic Dysfunction Test (CADT) is a questionnaire developed to evaluate the main symptoms of autonomic dysfunction and Kumamoto scale is a composite score entailing sensory disturbances, motor weakness, autonomic dysfunction and visceral organ impairment. Occurrence of orthostatic hypotension may be revealed by a tilt test. Scores mostly developed for Charcot-Marie-Tooth (CMT) disease have been applied to TTR-FAP, such as Neuropathy Impairment Score (NIS) with subscales and CMT Neuropathy score (CMTNS) and CMT Examination score (CMTES). The 6-minute walk test (6MWT), largely used in other neuromuscular diseases and evaluating the global and integrated responses in walking including the pulmonary and cardiovascular systems and neuromuscular units, is currently under validation in Italian TTR-FAP patients. Measurement of Modified Body Mass Index is useful in monitoring malnutrition and gastrointestinal dysfunction. Clinicians must also focus on possible renal and ocular involvement. Last but not the least, constant heart evaluation includes blood troponins and N-terminal pro-hormone brain natriuretic peptide (NT-proBNP), ECG, 2D-echocardiography with Doppler examination and deformation imaging, cardiac magnetic resonance imaging (MRI) and Technetium-99m-diphosphonate (99mTc-DPD) scintigraphy.

## Oral presentations

### Topic: Epidemiology, genotypes, phenotypes, and natural history of all types of ATTR

#### O1 Transthyretin amyloidosis cardiomyopathy: genotype, phenotype, quality of life and outcome in 997 patients in the UK

##### Thirusha Lane, Cristina Quarta, Joan Caringal-Galima, Dorota Rowczenio, Janet Gilbertson, David Hutt, Babita Pawarova, Tamer Rezk, Ana Martinez De Azcona Naharro, Sarah Anderson, Marianna Fontana, Daniel Knight, Carol Whelan, Ashutosh Wechalekar, Helen Lachmann, Philip Hawkins, Julian Gillmore

###### National Amyloidosis Centre, University College London, London, UK

####### **Correspondence:** Thirusha Lane (t.lane@ucl.ac.uk)

**Background**

Recent developments in non-invasive diagnostic imaging techniques have revealed a greater prevalence of transthyretin amyloid cardiomyopathy (ATTR-CM) than previously recognised. We sought to characterise the cohort of patients with ATTR-CM under the care of the UK National Amyloidosis Centre (NAC).

**Materials and methods**

We conducted a mixed retrospective and prospective study of patients with ATTR-CM, examining characteristics at diagnosis and at 6 to 12 month follow-up, including assessment of functional status and quality of life (QoL).

**Results**

Nine hundred ninety-seven patients with ATTR-CM attended the NAC between July 2005 and July 2017. 862 were male (86%). Median age at the diagnostic visit (case 1) was 77 (interquartile range, IQR, 71-82) years. 681 patients (68%) were wild-type (ATTRwt), 316 (32%) hereditary (ATTRv). The commonest variants in the ATTRv population were V122I (*n*=201; 64% of the ATTRv cohort), T60A (96; 30%) and S77Y (14; 4%). Within the ATTRwt cohort, 640 (94%) were male, whilst in the ATTRv cohort, 222 (70%) were male. Median age of ATTRwt patients at diagnosis was 79 (73-83), compared with 73 (67-97) in ATTRv patients (*p*<0.0001). Patients with the V122I variant, although similar in age to the ATTRwt patients, at case 1, at 77 (72-80), appeared to be functionally worse at baseline: 28% of V122I patients were too unwell to perform the baseline six-minute walk test (6MWT) compared with 15% of ATTRwt; the median distance walked by those able was 241 (147-349) metres by V122I versus 340 (230-414) metres by ATTRwt. Analysis of SF-36 data revealed that V122I patients had worse limitation of physical and social function, greater symptom burden and poorer overall QoL than ATTRwt at case 1. Survival differed between the cohorts: 57 months from baseline in ATTRwt versus 43 in ATTRv (*p*=0.0047) and 31 in V122I (*p*<0.0001). Baseline factors prognostic of survival included physical impairment, cardiac biomarkers and renal function.

**Conclusions**

Patients with ATTR-CM are already significantly functionally and socially impaired by the time they are diagnosed, indicating substantial diagnostic delay. Patients with ATTR-CM associated with the V122I variant appear to have the most severe phenotype at diagnosis, and poorer outcomes. In this era of promising novel therapies for ATTR amyloidosis, population screening studies may be useful to establish the diagnosis earlier in the disease course.

#### O2 A new staging system for cardiac ATTR amyloidosis

##### Julian D. Gillmore^1^, Thibaud Damy^2^, Marianna Fontana^1^, Matthew Hutchinson^1^, Helen J. Lachmann^1^, Ana Martinez Naharro^1^, Candida Quarta^1^, Tamer Rezk^1^, Carol J. Whelan^1^, Esther Gonzalez-Lopez^1^, Thirusha Lane^1^, Janet A. Gilbertson^1^, Dorota Rowczenio^1^, Aviva Petrie^1^, Philip N. Hawkins^1^

###### ^1^UCL, London, UK; ^2^Henri Mondor Teaching Hospital, Creteil, France

####### **Correspondence:** Julian D. Gillmore (j.gillmore@ucl.ac.uk)

**Background**

Cardiac transthyretin (ATTR) amyloidosis is an increasingly recognised, progressive and fatal cardiomyopathy, the natural history of which remains unclear. We sought to establish and validate a new prognostic staging system applicable to patients with both wild-type (ATTRwt) and hereditary variant (ATTRv) ATTR amyloid cardiomyopathy.

**Materials and methods**

Eight hundred and sixty-nine patients with cardiac ATTR amyloidosis (553 with ATTRwt and 316 with ATTRv) attending the UK National Amyloidosis Centre, were stratified into 3 disease stages at baseline on the basis of cut points in two universally measured biomarkers, NT-proBNP and estimated GFR (eGFR). Stage I was defined as NT-proBNP ≤3000 ng/L and eGFR ≥45 ml/min, Stage III was NT-proBNP >3000 ng/L and eGFR <45 ml/min; the remainder were Stage II. The staging system was validated in a cohort of 318 patients with cardiac ATTR amyloidosis from France.

**Results**

Median survival among 393 (45%) Stage I patients was 69.2 months, 334 (38%) Stage II patients was 46.7 months, and 142 (16%) Stage III patients was 24.1 months (*p*<0.0001). After adjusting for age, compared to Stage I the hazard ratio (HR) for death for Stage II was 2.05 (CI: 1.54-2.72, *p*<0.001) and for Stage III was 3.80 (CI: 2.73-5.28, *p*<0.001). HRs and statistical significance were little altered by TTR genotype, and were maintained in the validation cohort.

**Conclusions**

This simple, universally applicable staging system stratifies patients with both ATTRwt and ATTRv amyloid cardiomyopathy into prognostic categories. It will be of value in the design of forthcoming clinical trials of novel amyloid-specific therapies.

#### O3 Transthyretin familial amyloid polyneuropathy (ATTR V30M) in Cyprus: an updated epidemiological, clinical and genetic study

##### Savanna Andreou^1^, Elena Panayiotou^1^, Kyriaki Michailidou^2^, Panayiota Pirpa^2^, Andreas Hadjisavvas^2^, Daniel Barnes^3^, Antonis Antoniou^3^, George Tanteles^4^, Theodoros Kyriakides^1^

###### ^1^Department of Neuropathology/ Neurology Clinic A, The Cyprus Institute of Neurology & Genetics, Nicosia, Cyprus; ^2^Department of Electron Microscopy/Molecular Pathology, The Cyprus Institute of Neurology & Genetics, Nicosia, Cyprus; ^3^Centre for Cancer Genetic Epidemiology, Department of Public Health and Primary Care, University of Cambridge, Cambridge, UK; ^4^Clinical Genetics Clinic, The Cyprus Institute of Neurology & Genetics, Nicosia, Cyprus

####### **Correspondence:** Savanna Andreou (savannaa@cing.ac.cy)

**Background**

ATTR V30M is a lethal autosomal dominant sensorimotor and autonomic neuropathy caused by amyloid deposition. Amyloid consists of aggregated misfolded TTR monomers with the V30M mutation. ATTR V30M neuropathy has been described in many endemic foci such as Portugal, Sweden, Japan and Cyprus. The varied age of onset in patients with ATTR V30M varies in different foci and the mechanism behind it is still unknown. The modifying role of the complement protein C1q has recently been identified as a possible modifier. The current study aims to provide an updated epidemiological status of ATTR V30M in Cyprus and investigate the modifying effect of the C1q component on the patients’ age of onset.

**Materials and methods**

Demographic data were collected from the patients’ files at the Cyprus Institute of Neurology and Genetics, where all Cypriot ATTR V30M patients are diagnosed by clinical and genetic testing. DNA was collected and a candidate gene approach was performed for 21 C1q tagging SNPs: 19 SNPs were assessed by allelic discrimination using real-time PCR and 2 SNPs using conventional PCR/sequencing protocol.

**Results**

Eighty-two patients (deceased included) have been diagnosed with ATTR V30M in Cyprus so far, with the mean age of onset being 44.5±15. On the 31st of December 2015, the prevalence was 6.61/100,000, while the mean incidence between 2003 and 2015 was 0.32/100,000. Anticipation analysis indicated that the disease’s onset is on average 9.57 years earlier in offspring than in manifesting parents. SNP analysis of 80 manifesting carriers and 19 non-manifesting carriers revealed rs672693 (A>G) with minor allele frequency 0.38 and hazard risk 1.63 (CI: 1.15-2.30) having the most significant modifying effect on age of onset. When analyzing only the manifesting carriers, the most significant modifying effect comes from rs665691 (G>C) with minor allele frequency 0.38 and hazard risk 1.54 (CI: 1.17-2.03).

**Conclusions**

The current study has provided updated epidemiologic data of ATTR V30M neuropathy in Cyprus and identified two C1q tagging that influence age of onset.

#### O4 TTR FAP in Central and Eastern Europe

##### Ivailo Tournev^1^, Yesim Parman^2^, Sevim Erdem-Ozdamar^3^, Marta Lipowska^4^, Janez Zidar^5^, Menachem Sadeh^6^, Daniel Coriu^7^, Dijana Plaseska – Karanfilska^8^, Enver Bogdanov^9^, Olga Zinovyeva^10^, Mariana Gospodinova^11^, Stayko Sarafov^1^

###### ^1^Expert TTR FAP Center, UMBAL Aleksandrovska; Department of Neurology, Medical University, Sofia, Bulgaria; ^2^Neurology Department, Istanbul Medical Faculty, Istanbul University, Istanbul, Turkey; ^3^Neurology Department University Neuromuscular Disease Research Laboratory, School of Medicine, Ankara, Turkey; ^4^Department of Neurology, Medical University of Warsaw, Warsaw, Poland; ^5^Institute of Clinical Neurophysiology, University Medical Centre Ljubljana, Ljubljana, Slovenia; ^6^Department of Neurology, Edith Wolfson Medical Center, Holon, Israel; ^7^Department of Hematology; University of Medicine "Carol Davila", Bucharest, Romania; ^8^Research Centre for Genetic Engineering and Biotechnology "Georgi D. Efremov", within the Macedonian Academy of Sciences and Arts – Skopje, Macedonia; ^9^Department of Neurology, Kazan State Medical University, Kazan, Russian Federation; ^10^Department of nervous diseases and neurosurgery, I.M. Sechenov First Moscow State Medical University, Moscow, Russian Federation; ^11^Medical Institute of Ministry of Inferior, Sofia, Bulgaria

####### **Correspondence:** IvailoTournev (itournev@emhpf.org)

**Background**

TTR amyloidosis was first identified in Northern Portugal, where it was found to be associated with a Val30Met mutation of the TTR gene. Portugal, Brazil, Japan, and Sweden are considered endemic regions. More than 120 different mutations were identified in the whole world. In the last ten years there is a big advance in identification of the disease in Central and Eastern Europe. The disease was found in Bulgaria, Turkey, Cyprus, Poland, Romania, Izrael, Slovenia, Macedonia, Kosovo.

**Materials and methods**

A survey was performed among reference centers for ATTR amyloidosis in central and eastern Europe countries to detect TTR-FAP cases.

**Results**

In Bulgaria, 112 TTR-FAP patients and 94 asymptomatic carriers from 80 affected families were identified with the following mutations: Glu89Gln – 63 families; Val30Met – 8 families; Ser77Phe – 6 families, Ser52Pro – 1 family and Gly47Glu – 2 Roma (Gypsy) families. In Turkey 28 TTR FAP patients were diagnosed – 19 in Istanbul and 9 – in Ankara. Val30Met – 11 patients. The following mutations were found: Glu89Gln – 5 patients; Gly47Glu – 4 patients, Gly53Glu – 3 patients; Thr49Ser – 2 patients; Glu54Lys – 2 patients and Glu54Gly – 1 patient. In Cyprus 50 patients and 140 asymptomatic carriers were identified. All of them have Val30Met mutation. In Poland the following TTR FAP mutations were found: V71A- 9 family members over four generations diagnosed with TTR-FAP; D38V - 1 patient; F33L - 1 patient and 1 carrier; Val30Met - 1 patient and 2 carriers; p.Glu81Lys mutation – one family. In Slovenia nine TTR FAP patients from 4 different families with four different rare mutations were found: Val122Ala, Val30Ala, Ile107Phe and Asp38Asn. In Romania four TTR-FAP cases were diagnosed. All of them have the same mutation - Glu54Gln. In Israel, 19 TTR FAP patients and 4 asymptomatic carriers were identified with the following mutations were identified: Ser77Tyr, Phe33Leu, Gly6Ser, Val32Ala andVal30Met. In the most patients the onset is in the sixth decade. In Russia several TTR families were found with Val30Met mutation and late onset of the disease. Macedonia and Kosovo several TTR FAP families were identified with Glu89Gln mutation.

**Conclusions**

There is a significant genetic and clinical heterogeneity of TTR FAP. Val30Met mutation was found in many countries in Central and Eastern Europe and it causes TTR FAP with late onset. Glu89Gln is specific regional Balkan-Mediterranean mutation, identified in Turkey, Bulgaria, Macedonia, Kosovo, Italy. Second regional Balkan-Mediterranean mutation, identified in Italy, Greece, Bulgaria. In Bulgaria the mutation was identified in Gypsies.

### Topic: New Tools for Diagnosis in ATTR Amyloidosis

#### O5 Mass spectrometry and immunohistochemistry are complementary techniques for typing of cardiac amyloid

##### Tamer Rezk, Janet Gilbertson, Nigel Rendell, Graham Taylor, Patrizia Mangione, Diana Canetti, Vittorio Bellotti, Philip Hawkins, Julian Gillmore

###### UCL Division of Medicine. National Amyloidosis Centre, London, UK

####### **Correspondence:** Tamer Rezk (t.rezk@ucl.ac.uk)

**Background**

Cardiac amyloidosis is a progressive and fatal condition the clinical management of which depends upon identifying the correct amyloid fibril precursor protein. Systemic chemotherapy is the treatment for cardiac AL amyloidosis but should be avoided in patients with cardiac ATTR amyloidosis, up to 20% of whom have a monoclonal gammopathy which is incidental to their amyloid. Proteomic analysis of amyloid can be used to determine the fibril protein when immunohistochemistry (IHC) is non diagnostic. We describe our single centre experience of proteomics in endomyocardial biopsies (EMB).

**Matherials and methods**

EMBs from 59 patients with suspected cardiac amyloid were stained with Congo red at the UK National Amyloidosis Centre (NAC) by the method of Puchtler et al along with a panel of monospecific antibodies against known amyloid fibril proteins, including TTR and immunoglobulin light chains. Proteomic analysis, on the Velos platform, was performed on laser micro-dissected tissue. Mass spectrometry data files were analysed using Mascot software.

**Results**

Fifty-six samples were Congo Red (CR) positive and 3 CR negative. Amyloid was confirmed on proteomics by presence of all 3 signature proteins (SAP, APOE and APOA4) in 44/56 (79%) and 2 signature proteins in 11/56 (20%); in 1 (2%) case proteomics failed to support CR staining, likely due to scanty available tissue. The proteomic ‘amyloid signature’ was not present in any of the 3 CR negative samples. 47/56 (84%) CR positive biopsies were definitively typed by IHC. However, there was no immunospecific staining (NISS) in 8/47 cases and in the remaining case, the tissue was too scanty for IHC. Proteomic analysis concurred with definitive IHC typing of amyloid in 44/47 (94%) cases but failed to support IHC results in 3/47 cases. Among 8 cases in which there was NISS by IHC, 3 were identified as AL (2 kappa, 1 lambda), 3 were ATTR, 1 was AApoA4 and one was not conclusively typed by proteomic analysis.

**Conclusion**s

Proteomic analysis and Congo red/immunohistochemical staining of EMBs are complimentary techniques for identifying and typing amyloid in EMBs. Both are associated with occasional false negative results but used together, presence and type of amyloid can be definitively determined in >96% of EMBs.

#### O6 T1, extracellular volume and rest myocardial blood flow mapping: a multiparametric mapping approach in ATTR cardiac amyloidosis

##### Ana Martinez-Naharro, Daniel Knight, Tushar Kotecha, Rohin Francis, Tamer Rezk, Cristina Quarta, Esther Gonzalez, Richa Manwani, Carol Whelan, Helen Lachman, Ashutosh Wechalekar, Julian Gillmore, Philip Hawkins, Marianna Fontana

###### National Amyloidosis Centre, UCL, London, UK

####### **Correspondence:** Ana Martinez-Naharro (a.naharro@ucl.ac.uk)

**Background**

Cardiac involvement is the main driver of outcome in systemic amyloidosis, but the relationship between amyloid deposits and cellular injury is not well understood. The simple explanation of physical, mechanical replacement of parenchymal tissue seems insufficient, and preliminary studies support the hypothesis that myocardial hypoperfusion could contribute to cell damage in amyloidosis. The aim of this study was: 1) To assess feasibility of fully automated pixel-wise rest myocardial blood flow (MBF) mapping in cardiac amyloidosis during routine clinical scans; 2) To assess the prevalence of myocardial hypoperfusion and correlation with amyloid deposits and disease severity.

**Materials and methods**

Patients with cardiac ATTR (n=28) amyloidosis and healthy volunteers (n=16) were recruited. All subjects underwent CMR at 1.5T (Siemens) with standard SSFP cine imaging, Phase Sensitive Inversion Recovery Reconstruction Late Gadolinium Enhancement (PSIR-LGE), T1 mapping, Extracellular Volume (ECV) mapping and rest MBF mapping.

**Results**

The pixel-wise MBF maps for all slices were generated automatically in all patients within 2.5 minutes after image acquisition. Myocardial perfusion was globally reduced in patients with cardiac amyloidosis compared to healthy volunteers (0.53±0.12ml/min/g vs 0.85±0.09ml/min/g, *p*<0.05). Myocardial perfusion inversely correlated with amyloid burden measured as extracellular volume fraction (r = -0.57, *p*<0.01) and with the transmurality of LGE (no LGE 0.84±0.19ml/min/g, subendocardial LGE 0.56±0.10ml/min/g and transmural LGE 0.51±0.14ml/min/g, *p*<0.01).

**Conclusions**

Myocardial perfusion can be measured in cardiac amyloidosis during routine clinical scans with fully automated MBF mapping. Myocardial hypoperfusion at rest is highly prevalent in subjects with cardiac amyloidosis, and correlates with the degree of amyloid infiltration and disease severity.

#### O7 Rethinking heart failure in TTR amyloidosis

##### Rodney Falk, Avinainder Singh

###### Cardiac Amyloidosis Program, Brigham and Women's Hospital, Boston, MA, USA

####### **Correspondence:** Rodney Falk (rfalk@bwh.harvard.edu)

**Background**

The mechanism of heart failure in TTR amyloidosis is complex, and not fully understood. Epidemiological data from patients with V122I indicate that this variant is associated with an increased risk of heart failure (HF), even though the typical phenotype of cardiac amyloid is rare. This suggests that the presence of a modest amount of amyloid, insufficient to cause significant LV thickening, may result in HF, perhaps requiring perhaps a “second hit”, such as hypertension.

It is well-recognized that patients with ATTRm neuropathy undergoing liver transplantation may have post-transplant progressive cardiac disease, presumably due to ongoing ATTRwt deposition in the heart, triggered by a seeding phenomenon. Whether seeding plays a role in other amyloid patients is unknown.

**Materials and methods**

We present four illustrative cases clinical cases, unrelated to liver transplantation for ATTRm, with unusual manifestations, that illustrate the two aspects of the disease noted above. These cases cast a broader light on heart failure mechanisms in this increasingly-recognized disease.

**Results**

Case 1: TTRwt on cardiac biopsy with an amyloidogenic TTRm -Error in pathological diagnosis or another explanation?

Case 2: A Thickening heart two decades after AL “cure” for AL nephropathy –

Case 3: HF with normal wall thickness and HFrEF - amyloid or not?

Case 4: AL amyloidosis in an African man destined for HF despite hematologic cure?

**Conclusions**

These patients highlight the importance of thinking outside the box when evaluating patients with potential cardiac amyloidosis and the potential role of a seeding phenomenon.

### Topic: Updates in the Treatment for ATTR Amyloidosis; Clinical Trials of Disease Modifying Therapies

#### O8 Safety and efficacy of inotersen in patients with hereditary transthyretin amyloidosis with polyneuropathy (hATTR-PN)

##### Merrill Benson^1^, Márcia Waddington-Cruz^2^, Annabel Wang^3^, Michael Polydefkis^4^, Violaine Plante-Bordeneuve^5^, John Berk^6^, Fabio Barroso^7^, David Adams^8^, Peter Dyck^9^, Thomas Brannagan^10^, Carol Whelan^11^, Giampaolo Merlini^12^, Morton Scheinberg^13^, Brian Drachman^14^, Stephen Heitner^15^, Isabel Conceição^16^, Hartmut Schmidt^17^, Giuseppe Vita^18^, Josep Maria Campistol^19^, Josep Gamez, Edward Gane^21^, Peter Gorevic^22^, Acary Oliveria^23^, Brett Monia^24^, Steven Hughes^24^, Jesse Kwoh^24^, Bradley Mc Evoy^24^, Brenda Baker^24^, Andrew Shenker^25^, Helen Millns, Rito Bergemann^26^, Elizabeth Ackermann^24^, Morie Gertz^9^, Teresa Coelho^27^

###### ^1^Indiana University School of Medicine, Indianapolis, IN, USA; ^2^Federal University of Rio de Janeiro University Hospital, Rio De Janeiro, Brazil; ^3^University of California, Irvine, CA, USA; ^4^Johns Hopkins University, Baltimore, MD, USA; ^5^CHU Henri Mondor, Creteil, France; ^6^Boston University, Boston, MA, USA; ^7^FLENI, Ciudad Autónoma De Buenos Aires, Argentina; ^8^CHU Bicetre, Universite Paris-Sud, Paris, France; ^9^Mayo Clinic, Rochester, MN, USA; ^10^Columbia University Medical Center, New York, NY, USA; ^11^UCL, National Amyloidosis Centre, London, UK; ^12^Centro Amiloidosi Fondazione IRCCS, Pavia, Italy; ^13^Associação de Assistência a Criança Deficien, Sao Paulo, Brazil; ^14^University of Pennsylvania, Philadelphia, PA, USA; ^15^Oregon Health and Science University, Portland, OR, USA; ^16^CHLN - Hospital de Santa Maria, Lisbon, Portugal; ^17^Universitatsklinikum Münster, Münster, Germany; ^18^A.O.U. Policlinico G.Martino, University of Messina, Messina, Italy; ^19^Hospital Clínic, Barcelona, Spain; ^20^Hospital Universitari Vall D’ Hebron, Barcelona, Spain; ^21^Auckland City Hospital, Auckland, New zealand; ^22^Mount Sinai Medical Center, New York, NY, USA; ^23^Universidade Federal de Sao Paulo, Sao Paulo, Brazil; ^24^Ionis Pharmaceuticals, Inc, Carlsbad, CA, USA; ^25^GlaxoSmithKline, Philadelphia, PA, USA; ^26^GlaxoSmithKline, London, UK; ^27^Centro Hospitalar do Porto, Porto, Portugal

####### **Correspondence:** Merrill Benson (mdbenson@iupui.edu)

**Background**

Transthyretin (TTR) is a liver-derived protein that functions to transport vitamin A and thyroxin to peripheral tissues. In patients with hereditary TTR amyloid polyneuropathy (hATTR-PN), point mutations in the TTR gene encode single amino acid substitutions that induce deposition of amyloid in various organs including peripheral and autonomic nerves. Amyloid deposition eventually leads to multi-organ failure, with life expectancy ~10 years from symptom onset. Despite the use of liver transplantation and small molecule TTR stabilizers, there remains a high unmet medical need for new therapies to treat all forms of ATTR. Inotersen is a generation 2+ antisense oligonucleotide (ASO) inhibitor of TTR protein production.

**Materials and Methods**

A randomized, double-blind, placebo controlled phase 3 study (NEURO-TTR, NCT01737398) of inotersen in patients with hATTR-PN was conducted at 24 sites world-wide (USA 48%, Europe 35%, South-America 17%). The trial design included two co-primary endpoints: change from baseline in the composite modified Neuropathy Impairment Score +7 (mNIS+7) and patient reported Norfolk Quality of Life Diabetic-Neuropathy (Norfolk QoL-DN) score. Eligible patients were adults who had Stage I (ambulant) or Stage II (ambulant with assistance) disease characterized by an NIS score of 10 to 130 at screening, documented TTR variant by genotyping and documented amyloid deposition by biopsy. Enrolled patients were randomized (2:1) to receive 300 mg weekly SC doses of inotersen, or placebo, for 15 months.

**Results**

One hundred seventy-two patients received at least one dose of study drug. At baseline, the study population was 69% male, had a mean age of 59 years (range 27, 81) and a mean NIS of 45 based on the average of two assessments. The study cohort included 27 TTR mutations with 52% of patients expressing the Val30Met mutation. Eighty percent of patients completed the 15-month treatment period. Inotersen-treated patients achieved statistically significant benefit compared to placebo for mNIS+7 (*p*<0.0001) and Norfolk QoL-DN (*p*=0.0006). Key safety findings were thrombocytopenia and renal dysfunction in some patients. More than 95% who completed treatment have participated in the open-label extension study.

**Conclusions**

Inotersen demonstrated significant benefit on both primary clinical endpoints of neurological disease progression and quality of life in patients with hATTR-PN in the Phase 3 NEURO-TTR study.

#### O9 Patisiran, an investigational RNAi therapeutic for patients with hereditary transthyretin-mediated (hATTR) amyloidosis with polyneuropathy: results from the phase 3 apollo study

##### David Adams^1^, Alejandra Gonzalez-Duarte^2^, William O'riordan^3^, Chih-Chao Yang^4^, Taro Yamashita^5^, Arnt Kristen^6^, Ivaylo Tournev^7^, Hartmut Schmidt^8^, Teresa Coelho^9^, John L. Berk^10^, Kon-Ping Lin^11^, Peter J. Dyck^12^, Pritesh Gandhi^13^, Marianne Sweetser^13^, Jihong Chen^13^, Jared Gollob^13^, Ole Suhr^14^

###### ^1^National Reference Center for FAP, CHU Bicêtre, APHP, Le Kremlin-Bicêtre, France; ^2^National Institute of Medical Sciences and Nutrition - Salvador Zubiran, Mexico City, Mexico; ^3^eStudy Site, La Mesa, CA, USA; ^4^National Taiwan University Hospital, Taipei, Taiwan, Republic of China; ^5^Kumamoto University Hospital, Kumamoto, Japan; ^6^Heidelberg University Hospital, Heidelberg, Germany; ^7^University Multiprofile Hospital for Active Treatment, Sofia, Bulgaria; ^8^University Hospital Muenster, Muenster, Germany; ^9^Hospital de Santo António, Centro Hospitalar do Porto, Porto, Portugal; ^10^Amyloid Treatment and Research Program, Boston University, Boston, MA, USA; ^11^Taipei Veterans General Hospital, Taipei, Taiwan, Republic of China; ^12^Mayo Clinic Hospital, Rochester, MN, USA; ^13^Alnylam Pharmaceuticals, Cambridge, MA, USA; ^14^Umeå University Hospital, Umeå, Sweden

####### **Correspondence:** David Adams (david.adams@aphp.fr)

**Background**

hATTR amyloidosis is a multisystemic, rapidly progressive, life-threatening disease caused by a mutation in the TTR gene, resulting in deposition of amyloid fibrils in multiple organs. Heterogeneous clinical presentation of hATTR amyloidosis includes sensory, motor and autonomic neuropathy, as well as cardiac involvement, resulting in significant morbidity and mortality. Patisiran, an RNAi investigational agent, uses a naturally occurring pathway to target TTR mRNA in the liver. Previous Phase 2 OLE study data with patisiran in patients with hATTR amyloidosis with polyneuropathy showed that chronic dosing over 24 months was generally well-tolerated and resulted in a >80% sustained reduction of serum TTR and improvement in the mNIS+7 neuropathy impairment score. The Phase 3 APOLLO study aimed to further evaluate the efficacy and safety of patisiran in patients with hATTR amyloidosis with polyneuropathy.

**Methods**

Multi-center, international, randomized (2:1), double-blind, placebo-controlled study of patisiran 0.3mg/kg or placebo IV q3W in adult patients with hATTR amyloidosis with polyneuropathy (NCT01960348). Symptomatic patients with a neurological impairment score (NIS) of 5-130 were eligible. Select exclusion criteria: patients with prior liver transplant, PND score IV, and NYHA Class >2. Primary endpoint was a change from baseline at 18 months in the mNIS+7 neuropathy impairment score. Secondary endpoints included assessment of quality of life (Norfolk QOL-DN), motor strength (NIS-W), disability (R-ODS), gait speed (10-MWT), nutritional status (mBMI) and autonomic function (COMPASS-31).

**Results**

From Dec 2013 to Jan 2016, 225 patients were enrolled at 44 sites in 19 countries (EU: 51%; N. America: 21%; Asia-Pac: 20% and LatAm: 8%). Baseline demographics include median age: 62 years (range: 24-82); males: 74%; V30M: 42%, non-V30M: 57% (including 49 different TTR genotypes); and previous TTR tetramer stabilizer use: 53%. Measures of baseline disease severity included FAP Stage 1: 47%, FAP Stage 2: 52% and mean baseline mNIS+7: 78.6 (range: 8.0-165.0). Echocardiographic evidence of cardiac involvement was noted in 54% of patients.

**Conclusions**

APOLLO is the largest, controlled study of patients with hATTR amyloidosis with polyneuropathy and includes patients with a wide range of TTR genotypes and neuropathy severity, including >50% with cardiac involvement. Efficacy and safety results from this Phase 3 study will be presented.

#### O11 Proposal for a minimal neuropathy assessment protocol for ATTR amyloidosis in routine clinical practise

##### Mary M. Reilly^1^, Matilde Laura^1^, Andrea Cortese^1^, Michael Shy^2^, Davide Pareyson^3^

###### ^1^UCL Institute of Neurology, Queen Square, London, UK; ^2^University of Iowa, Iowa City, IA, USA; ^3^IRCCS Foundation, C.Besta Neurological Institute, Milan, Italy

####### **Correspondence:** Mary M. Reilly (m.reilly@ucl.ac.uk)

**Background**

Hereditary transthyretin (ATTR) amyloidosis is a dominantly inherited systemic disease caused by over 100 different pathogenic mutations in the transthyretin (TTR) gene. The multisystem involvement, the phenotypic heterogeneity and the variable disease course make the clinical evaluation of the disease and of its progression difficult. This is particularly true of the neuropathy. Although detailed protocols of the neuropathy have been developed for use in a clinical trial setting, there is no standardised agreed protocol for the neuropathy assessment in routine clinical practise. Many ATTR patients first present to a neurologist and early diagnosis is increasingly important with the advent of disease modifying treatments. Once the diagnosis is established it will become important to standardise the neuropathy evaluation so that decisions on the optimal time to initiate treatments can be made. In many countries it may be some time before a patient can be seen in a specialised amyloid centre so having accurate retrospective neuropathy assessments to help inform treatment decisions will be critical. This protocol needs to be simple enough to be performed in any neurological clinic.

**Materials and methods**

Based on current clinical practice, a literature review and our experience we propose the following minimal assessment protocol: Neuropathy Impairment Scale (NIS) and NIS-Lower Limb subscale (NIS-LL), Kumamoto scale, FAP stage, Norfolk Quality of life scale, FAP-R-ODS and the weighted Charcot-Marie-Tooth Neuropathy or examination score (CMTNSv2, CMTESV2).

**Conclusions**

We would like to discuss this protocol and agree an international standardised protocol going forward.

## Poster presentations

### Topic: Genotype, phenotype

#### P1 Genetic diagnosis in ATTR amyloidosis; a single UK centre 26 year experience

##### Dorota Rowczenio, Janet Gilbertson, Marianna Fontana, Ashutosh Wechalekar, Carol Whelan, Ana Martinez-Naharro, Candida Quarta, Tamer Rezk, Esther Gonzalez-Lopez, Philip Hawkins, Julian Gillmore, Helen Lachmann

###### National Amyloidosis Centre, UCL Medical School, London, UK

####### **Correspondence:** Dorota Rowczenio (d.rowczenio@ucl.ac.uk)

**Background**:

ATTR amyloidosis is caused by a deposition of either wild-type (ATTRwt) or variant (ATTRv) TTR fibrils. More than 120 amyloidogenic TTR mutations have been described and typically these are rare, reported only in isolated kindreds. ATTRv can be associated with a range of organ involvement, particularly neuropathy and cardiomyopathy, whereas ATTRwt presents with restrictive cardiomyopathy in the elderly.

**Materials and Methods**:

This retrospective study includes all patients referred between 1991 and 2017 for assessment of known or suspected amyloidosis, who underwent TTR gene sequencing as part of their routine work up. Additional diagnostic procedures included immunohistochemistry, proteomics, echocardiography, CMR, DPD and SAP scintigraphy.

**Results**:

TTR gene was sequenced in 4457 patients, mutations were found in 724 (16%). The most prevalent variants were: V122I (40%); T60A (26%); V30M (17%). Less common mutations found in 125 cases (17%) included: S77Y (n= 16 cases); G47E (8); G47V (7); V20I (7); E89K (6); A97S (6); F33V, I68L and I107V (5 each); E89Q (4); I107F (4); G53E/A, V71A, S77F, A81V and H90D (3 each). 27 variants were found in ≤ 2 individuals. 16 patients were homozygous: 13 had V122I (all Afro-Caribbean); and single cases for V30M, T60A and D38V (Greek, Irish and Ghanaian ancestry respectively). 557 patients (94% male), were diagnosed with ATTRwt. The protective TTR substitution T119M was found in 11 subjects (allele frequency 0.12%). None had ATTR amyloidosis; 8 had AL lambda, 1 AL kappa, and in 2 there was no evidence of amyloid. 5 patients (all Afro-Caribbean) diagnosed with AL amyloidosis (4 lambda, 1 kappa) carried V122I. 4 of these patients died of cardiac amyloidosis between the ages of 46 and 55 years.

**Conclusions**:

Improvements in diagnostics resulted in an increase in referrals with ATTR type amyloid, now accounting for 23.7 % of new cases seen in the last 2 years of whom 69.3% were TTRwt. Of our TTRv cohort V122I was the commonest variant, almost always found in patients of African ancestry; T60A was the second commonest identified largely in patients of Irish ancestry; whilst V30M was found in a heterogeneous group originating from Cyprus, Greece, Portugal, Sweden, UK and Spain. Our findings of TTRv in 16% of screened patients highlights the need for genetics in the routine evaluation of suspected ATTR amyloidosis but the incidental finding of variants in cases of AL amyloidosis shows the importance of interpreting genetics in context.

#### P2 Clinical–genetic correlations in Bulgarian TTR– FAP cohort – ten years observations

##### Stayko Sarafov^1^, Mariana Gospodinova^2^, Andrey Kirov^3^, Tihomir Todorov^4^, Teodora Chamova^1^, Albena Todorova^4^, Ivaylo Tournev^1^

###### ^1^Expert TTR FAP Center, UMBAL Aleksandrovska, Sofia, Bulgaria; ^2^Medical Institute of Ministry of Inferior, Sofia, Bulgaria; ^3^IMDL Genome Centre Bulgaria, Sofia, Bulgaria; ^4^Genetic Medico-Diagnostic Laboratory “Genica”, Department of Medical Chemistry and Biochemistry, Medical University Sofia, Sofia, Bulgaria

####### **Correspondence:** Stayko Sarafov (stayko_sarafov@abv.bg)

**Background**

To present clinical-genetic correlations in Bulgarian TTR-FAP cohort according to: patient’s ethnicity, individual mutation, clinical phenotype, positive familial history, average age of onset and range, average survival and range, male/female ratio.

**Materials and methods**

Glu89Gln in 64 families, Val30Met in 9, Ser77Phe in 7, Gly47Glu in 2 gipsy families, Ser52Pro in 1. Salting out DNA extraction from whole EDTA blood samples from our patients. Sanger sequencing of targeted part (usually including exons 2 and 3) of TTR gene.

**Results**

Glu89Gln: mean age of onset - 52,7 years for both sexes, m/f ratio 1:1,2; mixed phenotype: onset with PNP+CTS in a ~2/3, cardiac onset in a ~1/3, GI onset in ~ 7-10%. In a 4/5 of the cases the onset is after the age of 45-50. M/f survival is the same. Familial history (+) in 85%.

Ser77Phe: clinically mixed phenotype similar to Glu89Gln, average age of onset - 57,6 years. Mostly men are affected. Familial history (+) in 75%

Val30Met: highest average of onset – 66 years, not rare after age of 70. Phenotype at the onset is usually Polyneuropathy. Mostly men are affected. Familial history is uncertain, (+) in no more than 25%, usually presented as “sporadic”.

Ser52Pro – one female, age of onset 44,2 years; mixed phenotype PNP onset with early kidney involvement.

Gly47Glu (in gipsies) – mean age of onset 35,3 years, mixed phenotype with GI onset, early kidney involvement.

The average survival for all mutations is 8,3; range 7,9-9,4 except for Gly47Glu – 5 years.

**Conclusions**

The different mutations expresses less or more mixed phenotype at the onset. The average age and rage of onset are connected. Age of onset differs – earliest in Glu47Gly and latest in Val30Met. Family history appears to be inversely related to the age of onset. The average survival is lowest in Glu47Gly and the same for the other mutations. Males appear to have more severe phenotype and were affected more than females except for Glu89Gln - preliminary data. Val30Met is „rare“, due to a “specific” clinical phenotype possible explanation for a small number of patients.

#### P3 Genotypic and phenotypic presentation of Val30Met mutation in a Turkish cohort

##### Arman Çakar, Erdi Sahin, Hacer Durmus-Tekçe, Yesim Parman-Gülsen

###### Neurology Department, Istanbul University, Istanbul Faculty of Medicine, Istanbul, Turkey

####### **Correspondence:** Arman Çakar (arrmmaan@hotmail.com)

**Background**

Transthyretin-related familial amyloid polyneuropathy (TTR-FAP) is an autosomal dominant disorder caused by mutations of the transthyretin (TTR) gene. Mutant TTR protein dissociate from its native tetramer form to monomer form and aggregate in several tissues and organs. More than 100 different mutations of the transthyretin gene are identified worldwide, but still the first described Val30Met is the most common one. Although, length dependent sensory-motor polyneuropathy accompanied by autonomic involvement is the most common feature of TTR-FAP, various clinical presentations can be observed.

**Patients and methods**

Herein, we studied clinical, and genetic characteristics in eight patients from seven unrelated families with Val30Met mutation in TTR gene from a non-endemic region.

**Results**

There were 1 female and 7 male patients. Mean age of onset was 50.1±11.2 (ranges 37-66) years. Mean follow-up duration was 6.1±2.1 years. Mean presenting symptom was paresthesiae in lower limbs, whereas one patient had asymmetric numbness and pain in upper limbs. Two patients were homozygous for Val30Met. Age at onset and the severity of the neuropathy were not different from the heterozygotic patients. Ocular involvement with vitreous opacities was only observed in a homozygous patient. Cardiac symptoms were rare at the presentation, but 5 patients demonstrated evidence of amyloid cardiomyopathy during the follow-up. Mean disease duration at the time of the observation of evident cardiomyopathy signs was 4.5±2.7 years after neuropathy. Low creatinine clearance and proteinuria suggesting renal involvement was observed in one patient but kidney biopsy could not be performed. Two patients died during the follow-up due to cardiac dysfunction.

**Conclusions**

TTR-FAP is a rare, but progressive and fatal, systemic disease with predominant neurologic involvement. Our patients suggest TTR-FAP that patients with Val30Met mutation can exhibit clinical heterogeneity.

#### P4 Transthyretin amyloidosis in Slovenia

##### Janez Zidar (janez.zidar@kclj.si)

###### Institute of Clinical Neurophysiology, Ljubljana, Slovenia

**Background**

Two years ago we reported on two families with 4 members having transthyretin amyloidosis (TTR-A) and one sporadic patient with the senile systemic TTR-A that were at that time ascertained in Slovenia. During the last two years two new families with 5 affected members were found and two new cases with the senile systemic TTR-A

**Cases report**

The index case in the first family presented with cardiomyopathy and polyneuropathy at the age of 54. In addition he had vitreous opacities in the right eye. The patient died the day after liver transplantation. The TTR mutation was characterised as Val122Ala. His son, who was at that time of diagnosis asymptomatic, was found to be the disease carrier. He is, unfortunately lost for follow-up.

The disease of the second family’s index case started with pain in his feet, orthostatic hypotension, sexual impotence, diarrhoea alternating with constipation, and weight loss. Later on he also noticed weakness in his lower limbs. The Val30Ala mutation was found. Except to the polyneuropathy, which is in his case rapidly progressive despite therapy with tafamidis, no other organs are affected. His sister is asymptomatic but is having electrophysiological signs of motor polyneuropathy and cardiac vagal denervation. She is taking tafamidis and her condition is stable during the last 2 years.

Sporadic female patient from the third family was diagnosed only 8 years after the disease onset. She has rather rapidly progressive form of polyneuropathy with signs of the autonomic, sensory and motor nerve fibres involvement. The mutation found in this family is Ile107Phe. At the time of diagnosis this lady was already wheel-chair bound and did not qualify for the treatment with tafamidis.

The fourth family is family with cardiac form of the disease. Three affected members are already deceased. The fourth, the index case, presented with cardiac failure at the age of 45 and is currently doing well on medical treatment. Asp38Asn mutation was found as the cause.

**Conclusions**

In the last few years 9 patients with TTR-A from 4 different families with four different rare mutations were found in Slovenia. In addition 3 male patients with wild type TTR –A were discovered. We believe this was instigated by the increased awareness of the disease, especially amongst neurologists and cardiologists.

**Consent to publish**

Written informed consent was obtained from the patients involved in this study.

#### P5 

**Withdrawn**

#### P6 Late-onset transthyretin familial amyloid polyneuropathy: characterization of Brazilian subjects from the THAOS registry

##### Márcia Waddington-Cruz^1^, Amanda Berensztejn^1^, Marcus V. Pinto^1^, Rajiv Mundayat^2^

###### ^1^Federal University of Rio de Janeiro, National Amyloidosis Referral Center, CEPARM, Rio De Janeiro, Brazil; ^2^Pfizer Inc., New York, NY, USA

####### **Correspondence:** Márcia Waddington-Cruz (mwaddingtoncruz@gmail.com)

**Background**:

Transthyretin familial amyloid polyneuropathy (TTR-FAP) is a severe small-fiber predominant polyneuropathy, typically emerging in patients’ third or fourth decade. More recently, a late-onset form of TTR-FAP involving both large and small sensory fibers and more severe motor and cardiac involvement has been recognized. Little is known about the condition in many countries, including Brazil, despite increasing numbers of late-onset FAP patients being diagnosed. To improve characterization of late-onset TTR-FAP by comparing demographic and clinical characteristics of patients in Brazil with late-onset and early-onset TTR-FAP.

**Materials and Methods**:

Demographic and clinical data at the time of enrolment for Brazilian subjects with symptomatic Val30Met TTR-FAP were extracted from the ongoing, multinational, longitudinal, observational Transthyretin Amyloidosis Outcomes Survey (THAOS; cut-off date: January 30, 2017). Subjects were divided into those with symptom onset at age <50 years (early-onset), and at age ≥50 years (late-onset).

**Results**:

A total of 162 subjects with TTR-FAP were enrolled in Brazil; 148 had the Val30Met mutation and, of these, 96 were symptomatic. Late-onset subjects (*n*=25, 26.0%) had a longer time to diagnosis (mean 5.1 years compared to 2.8 years) and were more likely to be misdiagnosed (68% of the cases compared to 26.8%) than early-onset subjects. Clinically, subjects with late-onset tended to have more severe neurological impairment and more frequent cardiac involvement, as shown by NIS (mean Neurology Composite Score of 101 compared to 70) and cardiac measures (ECG abnormalities in 88.9 % in contrast to 59.4 %) (Echocardiogram IVS thickness >12 mm in 69.2% of the cases in contrast to none of the early onset group).

**Conclusions** :

The late-onset form of TTR-FAP is not unusual in Brazil, tending to be more difficult to diagnose and presenting with a more severe phenotype. Increased characterization may assist earlier recognition and improve patient outcomes.

#### P7 Amyloidosis research consortium cardiac amyloidosis survey: results from patients with ATTR amyloidosis and their caregivers

##### Isabelle Lousada^1^, Mathew Maurer^2^, Melissa Warner^1^, Spencer Guthrie^3^, Kristen Hsu^1^, Martha Grogan^4^

###### ^1^Amyloidosis Research Consortium, Newton, MA, USA; ^2^Columbia University - New York, NY, USA; ^3^Biopharma Strategic Consulting, Seattle, WA, USA; ^4^Mayo Clinic, Rochester, MN, USA

####### **Correspondence:** Isabelle Lousada (ilousada@arci.org)

**Background**

Cardiac amyloidosis is a severe disease that can lead to cardiac dysfunction and death. Both hereditary transthyretin (hATTR) amyloidosis and wild-type transthyretin (wtATTR) amyloidosis may result in cardiac amyloidosis. Initial symptoms are often nonspecific, causing delays in diagnosis until the disease is advanced. To understand delays and errors in the diagnostic pathway for ATTR cardiac amyloidosis.

**Materials and methods**

The Amyloidosis Research Consortium (ARC) developed an online survey, which was distributed to patient lists of ARC, the Amyloidosis Foundation, and Amyloidosis Support Groups in January 2017. The survey was designed for all forms of amyloidosis, but is limited to ATTR in the present analysis.

**Results**

In this subanalysis, 139 responders (76 patients, 63 caregivers) completed the survey for ATTR amyloidosis (88 hATTR, 51 wtATTR). Initial reported symptoms were most commonly shortness of breath, fatigue, and dizziness (Table 2). A majority of hATTR (52%) and wtATTR patients (65%) were diagnosed with carpal tunnel before their diagnosis of amyloidosis. Most wtATTR patients had solely cardiac involvement (53%), while nearly all hATTR patients had involvement of ≥1 other organ (94%), most commonly nerves (74%) (Table 1). Caregivers reported that 35% of wtATTR patients were diagnosed in <12 months from the start of symptoms, while this was true in only 14% of hATTR patients. 17% of all respondents reported visiting 5 different physicians before receiving the correct diagnosis (Table 1). 57% of hATTR and 39% of wtATTR patients received a misdiagnosis; of those 76% and 75%, respectively, received treatment for the misdiagnosed condition. While cardiologists were found to correctly diagnose the most patients (35% in hATTR, 78% in wtATTR), they were also found to miss the most (26% in hATTR, 31% in wtATTR) (Table 1).

**Conclusions**

This represents the first survey compiling caregiver and patient experiences with cardiac ATTR amyloidosis. Alignment of caregiver with patient responses validates our patient-directed research. hATTR and wtATTR patients experience some differences in disease presentation and diagnosis; however, similarities in the diagnostic journey are present. Patients frequently receive misdiagnoses and often receive incorrect treatment for the misdiagnosed condition. Disease awareness and understanding of common presenting symptoms, like carpal tunnel, is vital, especially among those to whom patients are initially referred.

**Table 1 (abstract P7).** Hereditary ATTR (hATTR) vs. Wild-type TTR (wtATTR) survey responses, % (*n*)^*^

What organs are involved besides the heart? ^+^						
	Kidney	Nerves	Liver	GI	Skin	
hATTR	18% (16)	74% (65)	25% (22)	45% (40)	11% (10)	
wtTTR	20% (10)	18% (9)	16% (8)	6% (3)	8% (4)	
Number of organs involved besides the heart						
	0	1	2	3	4	≥5
hATTR	11% (10)	31% (27)	35% (31)	15% (13)	6% (5)	2% (2)
wtTTR	53% (27)	30% (15)	8% (4)	8% (4)	2% (1)	0% (0)
What diagnostic procedures were done? ^+^						
	Heart biopsy	Fat pad biopsy	Rectal biopsy	Kidney biopsy	PYP/DPD	MRI
hATTR	35% (31)	59% (52)	14% (12)	7% (6)	23% (20)	50% (44)
wtTTR	76% (39)	27% (14)	4% (2)	2% (1)	18% (9)	55% (28)
How many different doctors were seen before diagnosis?						
	1	2	3	4	5 or more	
hATTR	23% (20)	13% (11)	13% (11)	16% (14)	23% (20)	
wtTTR	18% (9)	41% (21)	16% (8)	8% (4)	8% (4)	
What type of physician made the diagnosis?						
	Cardiologist	Hematologist	Internist	Neurologist	Nephrologist	
hATTR	35% (31)	5% (4)	6% (5)	17% (15)	2% (2)	
wtTTR	78% (40)	6% (3)	6% (3)	2% (1)	2% (1)	
What type of doctor made a misdiagnosis?^+^						
	Cardiologist	Hematologist	Internist	Neurologist	Nephrologist	GP
hATTR	26% (23)	3% (3)	10% (9)	5% (4)	8% (7)	16% (14)
wtTTR	31% (16)	2% (1)	6% (3)	2% (1)	2% (1)	6% (3)

^*^not all respondents answered all questions

^+^respondents could check all answers that applied

**Table 2 (abstract P7).** Patient vs. caregiver survey responses. % (*n*) ^*^

What organs are involved besides the heart? ^+^						
	Kidney	Nerves	Liver	GI	Skin	Other
Patient	11% (8)	50% (38)	17% (13)	26% (20)	5% (4)	43% (33)
Caregiver	28% (18)	57% (36)	27% (17)	37% (23)	16% (10)	16% (10)
What diagnostic procedures were done? ^+^						
	Heart biopsy	Fat pad biopsy	Rectal biopsy	Kidney biopsy	PYP/DPD	MRI
Patient	57% (43)	53% (40)	11% (8)	3% (2)	32% (24)	68% (50)
Caregiver	43% (27)	41% (26)	10% (6)	8% (5)	8% (5)	35% (22)
What were the presenting symptoms? ^+^						
	Shortness of breath	Fatigue	Edema	Dizziness	Fainting	Neuropathy
Patient	54% (41)	41% (31)	21% (19)	24% (18)	21% (16)	25% (19)
Caregiver	43% (27)	30% (19)	22% (14)	21% (13)	11% (7)	32% (20)
What type of physician made the diagnosis?						
	Cardiologist	Hematologist	Internist	Neurologist	Nephrologist	
Patient	61% (46)	7% (5)	7% (5)	13% (10)	1% (1)	
Caregiver	40% (25)	3% (2)	5% (3)	10% (6)	3% (2)	
What type of physician made a misdiagnosis? ^+^						
	Cardiologist	Hematologist	Internist	Neurologist	Nephrologist	GP
Patient	28% (21)	3% (2)	4% (3)	7% (5)	3% (2)	8% (6)
Caregiver	29% (18)	3% (2)	14% (9)	5% (3)	5% (3)	17% (11)

^*^ not all respondents answered all questions

^+^ respondents could check all answers that applied

### Topic: Phenotypic Variability

#### P8 Phenotypic variability of TTR Val122Ile mutation: a Caucasian patient with axonal neuropathy and normal heart after two years follow up

##### Claudia Stancanelli^1^, Luca Gentile^2^, Gianluca Di Bella^2^, Fabio Minutoli^3^, Massimo Russo^4^, Giuseppe Vita^2^, Anna Mazzeo^2^

###### ^1^Biomedical Department of Internal Medicine and Specialistic, University of Palermo, Palermo, Italy; ^2^Department of Clinical and Experimental Medicine, University of Messina, Messina, Italy; ^3^Department of Biomedical and Dental Sciences and of Morphological and Functional Images, University of Messina, Messina, Italy; ^4^Nemo Sud Clinical Centre, AOU Policlinico, Messina, Italy

####### **Correspondence:** Claudia Stancanelli (claudia.stancanelli09@gmail.com)

**Background**

Val122Ile is one of more than 100 mutations in transthyretin gene (TTR) that is associated with transthyretin-related hereditary amyloidosis (ATTR) with a prevalence as high as 3.9% in Afro-Americans. It has, as main clinical expression, a hypertrophic restrictive cardiomyopathy with mild or no neurological symptoms. A prospective study performed in an American urban population showed only two of 453 DNA samples from Caucasian newborns being positive for Val122Ile (0.44%). Significant ethnic differences are present in the TTR gene, and some differences may affect its function. In particular, the non-coding variants potentially associated with regulatory function showed a significant diversity between African and non-African individuals.

**Case report**

We report the first Caucasian patient carrying Val122Ile mutation with an axonal neuropathy and no cardiac involvement. The patient came to our attention 2 years ago complaining of numbness in the lower limbs and poor balance. Family history was negative for neuromuscular diseases. He had been diagnosed with a normal pressure hydrocephalus at age 69. At the first evaluation, his neurological examination showed deep tendon areflexia, hypoaestesia, and hypopallestesia on hands and feet. Neurophysiological study showed a significant reduction of compound motor and sensory action potentials (SNAP) on the peroneal and ulnar nerve and absent sural SNAP indicating an axonal neuropathy. After 1 year, we reviewed the patient clinically because of a rapid worsening of gait, so that he required a stick to walk. He underwent a sural nerve biopsy that showed loss of axonal fibers, negative at Congo Red staining. Since our region Sicily is endemic for late-onset FAP due to Phe64Leu mutation, we performed TTR genetic analysis. Surprisingly, the patient carried Val122Ile mutation. In two years follow-up ECG, cardiac ultrasound and MR remained normal and (99m)Tc-DPD scintigraphy did not detect any heart uptake after 1 and 2 years.

**Conclusions**

Our report confirms the heterogeneity in the genotype–phenotype correlation of ATTR, suggesting that other factors may interact with disease causing mutations. The absence of cardiac involvement suggests the possible understimation of Val122Ile mutation in non-African population. In addition, with the recent discovery of gene modifiers and the “cis-regulatory” hypothesis in Val30Met, we have now greater needs for TTR genetic analysis than in the past as well as more uncertainties in family genetic counseling

**Consent to publish**

Written informed consent was obtained from the patients involved in this study.

#### P9 The c.337-18 G>C mutation in the transthyretin gene is amyloidogenic

##### Sami Khella, Patricia Divito, Brian Drachman

###### University of Pennsylvania, Philadelphia, PA, USA

####### **Correspondence:** Sami Khella (sami.khella@mail.med.upenn.edu)

**Background**

To describe a previously unreported amyloidogenic autonomic neuropathy in a patient with a single point polymorphism (SNP) in the transthyretin (TTR) gene. Hereditary amyloid neuropathy is most often due to a SNP in the TTR gene. There have been a number of variants of unknown significance (VUS) in that gene. Most recently, a report of patients with combined autonomic and small fiber neuropathy had an association with non-amyloidogenic c.337-18 G>C mutation in the TTR gene.*

**Case report**

Case presentation from an amyloid referral practice in a large university. 74 year old African American man with progressive severe orthostatic hypotension and mild sensory motor neuropathy; both have been present 2 years. He had a history of long standing well controlled diabetes mellitus (recent HbA1C 7.2%) and a remote history of non-hodgkins lymphoma (2008). A fat pad aspirate revealed apple green birefringent material on congo red staining. Neither a skin nor an open fat biopsy showed similar findings. Genetic testing revealed c.337-18 G>C mutation in the TTR gene.

**Conclusions**

We describe a patient with a c.337-18 G>C mutation, a severe progressive autonomic and somatic neuropathy and evidence of amyloidosis. It is possible that this previously considered VUS affects the TTR gene function and results in amyloidosis.

**Consent to publish**

Written informed consent was obtained from the patients involved in this study.

*Levine, TD; Bland RJ: Incidence of Nonamyloidogenic Mutations in the Transthyretin Gene in Patients with Autonomic and Small Fiber Neuropathy. Muscle Nerve 14 JUN 2017, DOI: 10.1002/mus.25701

#### P11 A Val30Met sporadic familial amyloid polyneuropathy case with atypical presentation: upper limb onset of symptoms

##### Erdi Sahin, Yesim Parman, Hacer Durmus-Tekce, Arman Cakar

###### Neurology Department, Istanbul University, Istanbul Faculty of Medicine, Istanbul, Turkey

####### **Correspondence:** Erdi Sahin (erdisahin@gmail.com)

**Background**

Transthyretin-related familial amyloid polyneuropathy (TTR-FAP) is an autosomal dominant disorder caused by the mutations of the transthyretin (TTR) gene. Length dependent axonal sensory-motor and autonomic polyneuropathy is the hallmark feature of TTR-FAP hence, lower limb sensory symptoms are generally the initial manifestations. Yet, Koike et al reported that 5 of 50 patients presented with upper limb sensory symptoms. Herein, we describe a patient with Val30Met mutation presented with asymmetrical upper limb symptoms which was not previously reported in non-endemic regions.

**Case report**

A 66-year-old male patient was admitted with 3-year history of progressive numbness and pain in right hand. He progressively deteriorated and his symptoms have spread to his left hand in several months. He was diagnosed as bilateral carpal tunnel syndrome and underwent bilateral surgical carpal tunnel ligament release. However, he gradually worsened with tingling and numbness spreading to his forearms followed by weakness in both hands without any significant lower limb symptoms. He has had recurrent constipation, orthostatism and impotence for three years. Two years after the onset of the upper limb symptoms he developed numbness in footpad, followed by pain and weakness in both legs. His medical history was significant for systemic hypertension and a myocardial infarction four years prior. In his neurologic examination, he had bilateral miosis, distal muscle weakness predominantly in upper and left-side with absent deep tendon reflexes, stocking and glove type hypoaesthesia and hypoalgesia, diminished vibration sensation. Nerve conduction studies revealed a demyelinating sensory and motor polyneuropathy syndrome accompanied by the signs of axonal loss. These findings were accompanied by sympathetic autonomic involvement. Echocardiography was performed which revealed secondary changes in the myocardium associated with amyloid deposition. His cardiac magnetic resonance imaging results correlated with the findings of echocardiogram. Minor salivary gland biopsy was performed and amyloid infiltration of the blood vessel wall was observed. Molecular analysis of TTR gene revealed heterozygous Val30Met mutation in exon 2.

**Conclusions**

TTR-FAP is a multisystemic and increasingly popular disease but still very difficult to recognize especially in non-endemic regions. Upper limb onset axonal polyneuropathy is a rare presentation in general practice and was not reported in TTR-FAP patients in non-endemic regions.

**Consent to publish**

Written informed consent was obtained from the patients involved in this study.

#### P12 Proteinuric renal phenotype in the Mallorca cohort of hereditary transthyretin amyloidosis patients

##### Asunción Ferrer-Nadal^1^, Mercedes Uson^2^, Tomás Ripoll^3^, Hernán Andreu^4^, Manuel Raya-Cruz^5^, Eugenia Cisneros-Barroso^5^, Juan Buades^5^

###### ^1^Nephrology Department. Hospital Son Llàtzer, Palma De Mallorca, Spain; ^2^Neurology Department. Hospital Son Llàtzer, Palma De Mallorca, Spain; ^3^Cardiology Department. Hospital Son Llàtzer, Palma De Mallorca, Spain; ^4^Gastroenterology Department. Hospital Son Llàtzer, Palma De Mallorca, Spain; ^5^Internal Medicine Department. Hospital Son Llàtzer, Palma De Mallorca, Spain

####### **Correspondence:** Asunción Ferrer-Nadal (asfnadal@yahoo.com)

**Background**

Kidney disease has been reported in patients with Transthyretin- Hereditary Amyloidosis (TTRhA) with different mutations over the last years. Portuguese group has published the largest series of patients with Val30Met mutation. In Portugal the most frequent type of renal manifestation is proteinuria with moderate or severe kidney disease. They identified one third of patients who developed proteinuria and 10% progressing to End Stage Renal Disease (ESRD). However, in Mallorca we observed 2 different phenotypes of renal disease in patients with Val30Met mutation. One, more common that is non-proteinuric and the other much less frequent proteinuric. Both types of phenotypes developed ESRD with need of renal replacement therapy (RRT) by different mechanisms.

**Materials and methods:**

Observational study of proteinuric patients in Mallorca Cohort with TTRhA (V30M).

**Results**

We present 7 patients (4,6%) with proteinuria more than 1 g/24h, median 2,5 g/24h (IQR 1,8-3,4). Mean age at the onset was 60,3 years (SD 8,7). 57% were female. 4 patients developed ESRD with need of RRT. The mean duration of renal replacement therapy was 1,9 years, with a 33% survival at two year treatment. 3 patients died at the mean age of 66,7 years (SD 5). 57% of patients had received a liver transplant and one liver-kidney transplant.

**Conclusions**

We have observed few patients with severe proteinuria (> 1g/24h), lower than other groups. It can be explained in part because of the high prevalence of patients with liver transplantation with anticalcineurin inhibitors drugs and high use of other common antiproteinuric therapies such as Angiotensin Converting Enzyme (ACE) inhibitors.

**References**

1. Lobato L. Portuguese-type amyloidosis (transthyretin amyloidosis, ATTR V30M). J Nephrol. 2003 May-Jun;16(3):438-42.

2. End-stage renal disease and dialysis in hereditary amyloidosis TTR V30M: presentation, survival and prognostic factors. L Lobato, I Beirão, M Silva, I Fonseca, J Queirós, G Rocha, A Morais Sarmento, A Soua, J Sequeiros. Amyloid 2004; Vol. 11: 27-37.

3. Lobato L, Rocha A. Transthyretin amyloidosis and the kidney. Clin J Am Soc Nephrol. 2012 Aug;7(8):1337-46.

#### P13 Ocular manifestations in S77T transthyretin-related familial amyloid polyneuropathy

##### Roxane Bunod^1^, Cécile Cauquil^2^, Halima Bourenane^2^, Emmanuel Barreau^1^, Marc Labetoulle^1^, David Adams^2^, Antoine Rousseau^1^

###### ^1^Department of Ophthalmology, NNERF, Bicêtre Hospital, APHP, DHU Vision & Handicaps, Paris-Sud University, Le Kremlin-Bicêtre, France; ^2^Department of Neurology, NNERF, Bicêtre Hospital, Assistance Publique - Hôpitaux de Paris, Paris-Sud University, Le Kremlin-Bicêtre, France

####### **Correspondence:** Roxane Bunod

**Background**

Transthyretin-related familial amyloid polyneuropathies (TTR-FAP) are associated with ocular manifestations including dry eye, amyloid deposits in the anterior chamber, secondary glaucoma and vitreous amyloid deposits and retinal angiopathy, which have been extensively described for V30M mutation. S77T is the second most frequent transthyretin amyloidogenic mutation in France after V30M and is associated with a severe neurologic and cardiac phenotype. However, ocular manifestations of this particular form of TTR-FAP have been scarcely described.

**Materials and methods**

This monocentric observational study was conducted at the French national reference center for TTR-FAP. Genetically confirmed S77T-TTR-FAP patients had complete neurologic and ophthalmologic evaluation. Sensorimotor polyneuropathy (SPN) was staged using the Polyneuropathy Disability (PND) score. Ophthalmological examination included best corrected visual acuity (BCVA), Schirmer test, intraocular pressure (IOP), slit lamp photographs, fundus examination with retinography. Medical and surgical treatments were analyzed for each patient.

**Results**

Eighteen S77T-TTR-FAP patients (11 males and 7 females), aged 31-71 years, (mean 58.2±11.1 years), all originating from France, were included. Symptomatic patients (*N*=15), had a mean PND of 1.9±1.3, and a delay between first symptoms and ophthalmological evaluation of 5.6±4.4 years. None of them presented with amyloid deposits in the anterior chamber, secondary glaucoma nor vitreous amyloid deposits. However, conjunctival lymphangiectasia were present in both eyes in 9 patients (50%) and associated with more severe neurologic disease (PND = 2.5±1.0 vs 1.2±1.2; *p*<0.05). Retinal ischemic amyloid angiopathy was found in one patient (5%). Dry eye was found in one third of patients.

**Conclusions**

Our results suggest that, unlike in V30M-TTR-FAP, anterior chamber deposits, secondary glaucoma and vitreous deposits do not occur in S77T-TTR-FAP. However, S77-TTR-FAP is associated with conjunctival lymphangiectasia, dry eye and amyloid angiopathy. Conjunctival lymphangiectasia occur in patients with severe neurologic disease and were not described in TTR-FAP associated with other TTR mutations, suggesting a genotype-phenotype correlation in TTR-FAP ocular manifestations, with conjunctival lymphangiectasia being a specific feature of S77T-TTR-FAP.

### Topic: Wild Type Ttr Amyloidosis

#### P14 The frequency of wild-type transthyretin amyloidosis in Russia according to the results of an autopsy study

##### Anzhelika Poliakova^1^, Evgeny Semernin^2^, Mariya Sitnikova^2^, Karapet Avagyan^2^, Roman Grozov^2^, Svetlana Pyko^3^, Alexandr Krutikov^2^, Viktoriya Davydova^2^, Tinatin Bezhanishvili^1^, Karina Khmelnitskaya^1^, Mihail Shavloskii^4^, Dmitrij Korzhevskii^4^, Alexandra Gudkova^1^

###### ^1^Pavlov First Saint Petersburg State Medical University, St. Petersburg, Russian Federation; ^2^Almazov Federal Medical Research Centre, St. Petersburg, Russian Federation; ^3^St. Petersburg Electrotechnical University “LETI”, St. Petersburg, Russian Federation; ^4^Federal State Budgetary Scientific Institution «Institute of Experimental Medicine», St. Petersburg, Russian Federation

####### **Correspondence:** Anzhelika Poliakova (lica.polyakova@mail.ru)

**Background**

Life-time diagnostics of wild type transthyretin amyloidosis (ATTR wt) is practically absent. At the same time, ATTR wt is an underestimated cause of morbidity and mortality, especially in the older age group. To study the frequency of detection, demographic parameters and morpho-functional features of TTR wt in patients with сhronic heart failure (CHF) I-IV functional classes and left ventricular hypertrophy ≥ 15 mm according to autopsy results.

**Materials and methods**

A retrospective analysis of the postmortem investigations of the patients of the cardiological department (*n* = 141) with the leading syndrome of CHF was carried out. The age was ≥ 69 years, men - 19%, women - 81%. All formalin-fixed paraffin blocks were stained with Congo red and viewed under polarized light. Immunohistochemical analysis was also performed using antibodies to AA-amyloid, transthyretin, prealbumin, kappa and lambda-light chains.

**Results**

Amyloid deposits were detected in old age and in long-livers, the average age was 91.25 ± 9.67 years, mainly in women due to the lower life expectancy of men. In patients with CHF of a different functional classes, associated with left ventricular hypertrophy (LVH), amyloid deposits occur in almost every fourth deceased (in 21% of cases) according to autopsy data. The amount of amyloid deposits in the myocardium were mostly small (56% of observations had amyloid deposits - (+) and 27% - (++)), a significant amount of amyloid was detected in 17% of cases (+++ - 7% and ++++ - 10 %). The presence of amyloid deposits did not significantly affect on the indices of myocardial hypertrophy, such as the thickness of the interventricular septum, left ventricle posterior wall and left ventricular mass index. In the presented cases, a focal amyloid deposits in the myocardium was observed which is typical for TTR wt, with that in 97% of cases amyloid deposits located in the interstitial area around the cardiomyocytes, and in 3% - exclusively around the vessels.

**Conclusions**

ATTR wt was detected in every fourth patient in a cohort of patients of old age and long-livers, predominantly in women (83%), and was not diagnosed during life. Typical morphological manifestations of ATTR wt are focal amyloid deposits, located mainly in myocardial interstitium.

#### P16 Machine learning predicts mortality better than biomarker staging in wild-type transthyretin cardiac amyloidosis

##### Avinainder Singh, Tara Mirto, Rodney Falk

###### Brigham & Women's Hospital, Boston, MA, USA

####### **Correspondence:** Avinainder Singh (asingh11@bwh.harvard.edu)

**Background.**

A biomarker-based staging system has recently been described for prognosis in wild-type transthyretin cardiac amyloidosis (ATTRwt). We validated this staging system in consecutive ATTRwt patients and compared its predictive accuracy to machine learning algorithms.

**Materials and methods.**

Clinical data was extracted from medical records of patients diagnosed with ATTRwt over the past 5 years. Diagnosis was based on technetium pyrophosphate scanning, or histopathological analysis of cardiac biopsy specimens. Data on death was collected from medical records as well as social security death index. The following machine learning algorithms were tested – Random Forest learner, AdaBoost, Naïve Bayes, Stochastic Gradient Descent (SGD). The model was built on a random sampling of 80% data, and tested on the remaining 20%, over 100 iterations. For comparison, a recently described staging system was used, which allots one point each for NT-proBNP >3000 pg/mL and troponin- T > 0.05 ng/mL, which results in the stages 1(0 points), 2 & 3 (2 points). The Area under the Curve (AUC) and the classification accuracy (proportion of outcome correctly predicted) were compared.

**Results**

Among 197 individuals with ATTRwt (mean age 76 ± 6 years, 6 women) 86 were in Stage 1 (44%), 66 in Stage 2 (33%) and 45 in Stage 3 (23%). There were 59 deaths (30%), with a median survival of 5.1 years. The staging system had an AUC of 0.70 (95% CI 0.62-0.77). All machine learning approaches performed better than clinical staging, except for AdaBoost (AUC – 0.71) (Table 1). Naïve Bayes had the highest AUC (0.86), whereas SGD had the highest classification accuracy (0.83).

**Conclusion**

Biomarker based staging is valid and has moderate accuracy for predicting mortality in patients with ATTRwt cardiac amyloidosis. However, machine learning algorithms can provide superior predictions compared with biomarker staging.

**Table 1 (abstract P16).** Predictive Accuracy of Machine Learning Approaches for Mortality in ATTRwt Cardiac Amyloidosis

Algorithm	AUC	Classification Accuracy
Random Forest	0.84	79%
Naive Bayes	0.86	77%
AdaBoost	0.71	74%
Stochastic Gradient Descent	0.80	83%
Clinical Staging	0.70	73%

#### P17 Characterization of wild-type transthyretin amyloidosis among women: preliminary results from an international multicenter study

##### Cristina C. Quarta^1^, Anna L. Tinuper^2^, Esther Gonzalez-Lopez^1^, Thirusha Lane^1^, Mathew Maurer^3^, Carol J. Whelan^1^, Arnt Kristen^4^, Rodney H. Falk^5^, Thibaud Damy^6^, Pablo Garcia-Pavia^7^, Giampaolo Merlini^8^, Claudio Rapezzi^2^, Julian D. Gillmore^1^, Philip N. Hawkins^1^

###### ^1^National Amyloidosis Centre, Division of Medicine, University College London, London, UK; ^2^Cardiology, Department of Experimental Diagnostic and Specialty Medicine (DIMES), Alma Mater Studiorum, University of Bologna, Bologna, Italy; ^3^Center for Advanced Cardiac Care, Columbia University College of Physicians and Surgeons, New York, NY, USA; ^4^Amyloidosis Center, Department of Cardiology, Heidelberg University, Heidelberg, Germany; ^5^Cardiac Amyloidosis Program, Division of Cardiology, Department of Medicine, Heart & Vascular Center, Brigham and Women's Hospital, Harvard Medical School, Boston, MA, USA; ^6^Centre de Reference National, Amyloses Cardiaques, UPEC, Créteil, France; ^7^Heart Failure and Inherited Cardiac Diseases Unit, Department of Cardiology, Hospital Universitario Puerta de Hierro Majadahonda, Madrid, Spain; ^8^Amyloidosis Research and Treatment Center, Fondazione IRCCS Policlinico San Matteo and University of Pavia, Pavia, Italy

####### **Correspondence:** Cristina C. Quarta (ccquarta@gmail.com)

**Background**

Over 90% of patients with wild-type transthyretin amyloidosis (ATTRwt) are reported to be male. This gender disproportion remains unexplained and little is known about the characteristics of female patients affected by ATTRwt. We aimed to to assess the clinical and instrumental findings of women with ATTRwt.

**Materials and methods**

We conducted a multicenter study of female patients diagnosed with ATTRwt at 8 internattional Amyloid Centres (London, Boston, New York, Paris, Pavia, Madrid, Heidelberg and Bologna).

**Results**

In 2005-2016, 97 women were diagnosed with ATTRwt. Exertional dyspnea was the main presenting symptom, with 75 patients (77%) showing NYHA class≥2. A history of carpal tunnel syndrome was reported in 45 (46%) patients.

Electrocardiographically, low QRS voltage was present in 16% of cases, I degree atrioventricular block in 17%, atrial fibrillation in 35%; left or right bundle branch block in 25%. Of the 62 patients with available scintigraphic data (either DPD or PYP or HMDP), 57 (92%) showed an intense cardiac uptake, with a visual score of 2 and 3 in 28 (45%) and 29 (47%) of cases, respectively.

Echocardiographically, patients showed a severe symmetric increase of the left ventricular (LV) wall thickness with no LV dilatation (LV end-diastolic diameter 40±6mm), preserved LV ejection fraction (54±12%) and diastolic dysfunction (E/E’ 17±7). Longitudinal systolic function was impaired (S’ 0.06 [0.04-0.07]m/s; global longitudinal strain -11±6%). Table 1 shows the main findings of the 97 female ATTRwt patients compared to 598 male patients diagnosed in the same period. Compared to men, women showed a better overall survival (Log-rank *p*=0.02).

**Conclusions**

This is the largest series of female ATTRwt patients ever studied. Compared to men, female patients were older, suggesting that women may get the disease slightly later in life, which could only partly explain the different incidence between men and women. Also, female patients showed less functional cardiac impairment and better survival. Our findings suggest the need of larger prospective studies involving more referral centers across the world in order to assess the real impact of gender and age on the incidence and pathophysiology of ATTRwt.

**Table 1 (abstract P17).** Main characteristics of female vs male patients with ATTRwt

	Female (*n*=97)	Male (*n*=598)
Age, yrs	79±8	77±7 ^*^
NT-proBNP, ng/L	3271 [1778-6151]ng/L	3087 [1624-5461]
LV wall thickness, mm	16±3	16±3
E/E’	17±7	19±7 ^*^
LV ejection fraction, %	54±12	47±13 ^*^
Global longitudinal strain, -%	-11±6	-9±6 ^*^

^*^ *p*<0.05

### Topic: Clinical Biology In ATTR Amyloidosis

#### P18 Serum transthyretin levels are significantly lower in Val122Ile cardiomyopathy compared with wild-type transthyretin cardiomyopathy

##### Avinainder Singh, Kevin Alexander, Rodney Falk

###### Brigham & Women's Hospital, Boston, MA, USA

####### **Correspondence:** Avinainder Singh (asingh11@bwh.harvard.edu)

**Background**

Recent data suggest that serum levels of biomarkers, such as prealbumin/transthyretin (TTR) levels or retinol binding protein-4, may assist in differentiating patients with Val122Ile amyloid cardiomyopathy from non-amyloid heart failure. Our objective was to assess whether TTR levels can help differentiate patients with Val122Ile cardiomyopathy from wild-type ATTR (ATTRwt) cardiomyopathy.

**Materials and methods**

Data from the Brigham & Women’s Hospital Cardiac Amyloidosis Program Database were retrospectively analyzed, querying patients with ATTR cardiomyopathy who had a serum TTR level measured at the time of diagnosis or initial visit. Diagnosis of amyloidosis was based on consensus criteria. The Val122Ile mutation was identified by genetic testing or mass spectrometry analysis of endomyocardial biopsy specimens. Patients with mutations other than Val122Ile were excluded. Receiver operator characteristic curves were constructed to identify optimal cut-offs. Cox proportional hazards modeling was performed to assess the prognostic impact of serum TTR concentration.

**Results**

The cohort consisted of 78 patients (mean age 74±6 years, 4 females), of whom 26 (33%) had Val122Ile cardiomyopathy. Serum TTR levels were significantly lower in patients with Val122Ile compared with ATTRwt (14.9±1.5 vs. 20.4±0.8; *p*=0.001). A serum TTR value less than 19.25 mg/dL had a sensitivity of 77% and specificity of 60% for Val122Ile cardiomyopathy (area under the curve=0.68). Low serum TTR was a predictor of mortality (HR 0.93, *p*=0.02). This effect was attenuated but remained significant after adjusting for NT-proBNP and troponin-T concentrations (HR 0.94, *p*=0.04). This association was stronger in those with ATTRwt (HR 0.93, *p*=0.06) compared to Val122Ile ATTR (HR 0.95, *p*=0.23).

**Conclusions**

Serum TTR levels are significantly lower in patients with Val122Ile ATTR than ATTRwt. Among patients with ATTRwt, a lower TTR level is associated with a worse prognosis. Mechanisms for a low serum TTR concentration in ATTRwt may include poor nutritional status or decreased TTR stability.

**References**

Arvanitis, Marios, et al. "Identification of Transthyretin Cardiac Amyloidosis Using Serum Retinol-Binding Protein 4 and a Clinical Prediction Model." JAMA Cardiology 2.3 (2017): 305-313.

#### P22 Immortalization of primary cells derived from attr patients

##### Paula Ballmaier, Christoph Niemietz, Sarah Guttmann, Sara Reinartz Groba, Andree Zibert, Hartmut Schmidt

###### Klinik für Transplantationsmedizin, Universitätsklinikum Münster, Münster, Germany

####### **Correspondence:** Paula Ballmaier (paulajohanna.ballmaier@ukmuenster.de)

**Background**

The penetrance, onset, symptoms, and prognosis differ between ATTR patients, even for the same mutant. Patient-specific mechanisms of amyloid fibril formation and clearance have been proposed to affect penetrance. Amyloid deposits preferentially occur in the extracellular matrix (ECM), largely produced by stromal cells. The impact of primary stromal cells derived from different TTR-FAP patients (e.g. presymptomatic vs. overt disease) for accumulation/internalization of TTR is not known. Urine-derived cells (UCs) are an easily attainable source of primary, fibroblast-like cells. The aim of this study is to establish immortalized primary cells from ATTR patients for the study of patient-specific amyloid deposits.

**Materials and Methods**

Retroviral transduction of UCs was performed using various gene combinations of transgenes. Cells were transduced with either hTERT/p53, CyclinD1/CDK4(R24C), and HPV16E6E7 or combinations thereof. The influence of gene transfer was assessed by determination of cell proliferation, mRNA expression (qRT-PCR) and protein expression (e.g. flow cytometry, immunofluorescence).

**Results**

Untreated or GFP transduced UCs underwent senescence after 5-10 days as determined by senescence-associated beta-galactosidase assay. In contrast, UCs could be cultured for several months (presently > 130 days) after HPVE6E7 gene transfer (*n*=3). UCs which were also transduced with CyclinD1/CDK4(R24C) showed accelerated cell growth as compared to other combinations and single HPVE6E7 expression. High expression levels of KRT7, FN1, SLC2A1, CD29 and CD44 where observed in the cells, whereas CD105 and CD90 expression was almost absent indicating epithelial and fibroblast cell marker expression. Immortalization did not affect marker epithelial/fibroblast marker expression as observed by immunofluorescence and qRT-PCR analysis. However, cell cycle regulator p21 was significantly downregulated after immortalization.

**Conclusion**

Our data indicate that immortalization of urine-derived cells is an excellent tool to generate primary ATTR fibroblast-like cell lines that are highly valuable for the molecular understanding of TTR ECM deposition using a patient-specific analysis.

#### P23 Mechano-enzymatic mechanism of transthyretin amyloidogenesis: long distance effects of optimally effective inhibitors

##### Vittorio Bellotti^1^, Patrizia P. Mangione^1^, Guglielmo Verona^1^, Alessandra Corazza^2^, Diana Canetti^1^, Julian D. Gillmore^3^, Philip N. Hawkins^3^, Graham W. Taylor^1^, Mark B. Pepys^1^

###### ^1^Wolfson Drug Discovery Unit, Centre for Amyloidosis and Acute Phase Proteins, UCL, London, UK; ^2^Department of Medical and Biological Sciences, University of Udine, Udine, Italy; ^3^National Amyloidosis Centre, UCL and Royal Free Hospital, London, UK

####### **Correspondence:** Vittorio Bellotti (v.bellotti@ucl.ac.uk)

**Background**

We have recently identified a new mechano-enzymatic pathway of transthyretin (TTR) amyloid fibrillogenesis, under physiological conditions, which is catalyzed by a selective proteolytic cleavage of the loop interconnecting the strands C and D. This model is consistent with the presence of the corresponding large TTR fragment in most of natural amyloid fibrils, particularly in cardiac amyloid deposits.

**Materials and methods**

We have used a range of physico-chemical techniques to monitor the effects of prototypic ligands bound by TTR on the structural dynamics, kinetics of proteolytic cleavage and fibrillogenesis of TTR.

**Results**

We observed that occupancy of the two symmetrical binding sites by known TTR stabilizers can modulate the susceptibility of the protein to the amyloidogenic proteolytic cleavage.

**Conclusions**

The most potent inhibition of mechano-enzymatic fibrillogenesis is achieved by ligands that simultaneously occupy both the binding sites and, uniquely by our family of bivalent ligands, also the central channel present at the dimer-dimer interface.

#### P24 Serum protein electrophoresis (spe): a review in hereditary transthyretin amyloidosis

##### Juan Buades, Manuel Raya-Cruz, Cristina Gallego-Lezaun, Asunción Ferrar-Nadal, Mercedes Uson, Antoni Figuerola, Cristina Descals, Joan Carles Montala, Tomas Ripoll, Juana Nuñez, Francisco Vega, Maria Ángeles Alonso, Hernán Andreu, Mateu Antonia, Eugenia Cisneros-Barroso

###### Hospital Son Llàtzer, Palma De Mallorca, Spain

####### **Correspondence:** Juan Buades (doctorjuanbuades@gmail.com)

**Background**

Proteins are made up of amino acids chains linked by peptide bonds. Proteins have in their structure carboxyl and amine groups that confer negative or positive charge to the proteins depending on the number of free acidic and basic amino acids, ternary and quaternary structure and pH and ionic strength of the media.

Proteins can be separated when exposed to an electric current because the speed of the movement depends on the charge of the protein and the strength of the electric field.

**Materials and methods**

Electrophoresis is a method of separating proteins based on their physical properties. Serum is placed on a specific medium, and a charge is applied. The net charge (positive or negative) and the size and shape of the protein commonly are used in differentiating various serum proteins.

**Results**

We have analyzed a cohort of 23 patients of AhTTR with the Val30Met mutation. We have found that 13 of 23 patients had serum protein electrophoresis alterations. Among them, 5 had a SPE image suggestive of chronic inflammation, 4 had acute inflammation profile, 3 a monoclonal band in the ɣ region and 1 showed hypogammaglobulinemia.

**Conclusions**

Classical immunofixation using IgG, IgM, IgA, kappa and lambda light chains antibodies is indicated to characterize the monoclonal band in the ɣ region. However, immunofixation failed to identify the composition of the band in 2 of the 3 patients.

### Topic: New Tools In ATTR Amyloidosis

#### P25 Diagnostic accuracy of 99mTc-DPD scintigraphy for detecting ATTR cardiac amyloid deposits

##### David F. Hutt^1^, Simona F. Grigore^1^, Joanne Page^1^, Maria Burniston^2^, Ann M. Quigley^3^, Daniel Knight^1^, Ana Martinez-Naharro^1^, Ashutosh D. Wechalekar^1^, Helen J. Lachmann^1^, Candida C. Quarta^1^, Tamer Rezk^1^, Richa Manwani^1^, Shameem Mahmood^1^, Sajitha Sachchithanantham^1^, Taryn Youngstein^1^, Carol J. Whelan^1^, Thirusha Lane^1^, Janet A. Gilbertson^1^, Dorota Rowczenio^1^, Julian D. Gillmore^1^, Marianna Fontana^1^, Philip N. Hawkins^1^

###### ^1^National Amyloidosis Centre, Division of Medicine, UCL, London, UK; ^2^Nuclear Medicine Department, Barts Health NHS Trust, London, UK; ^3^Nuclear Medicine Department, Royal Free London NHS Foundation Trust, London, UK

####### **Correspondence:** David F. Hutt (d.hutt@ucl.ac.uk)

**Background**

A number of authors have suggested that bone scintigraphy with planar quantitation is able to distinguish between cardiac AL and cardiac ATTR amyloidosis. We sought to investigate the diagnostic accuracy of 99mTechnetium labelled 3,3-diphosphono-1,2-propanodicarboxylic acid (99mTc-DPD) scintigraphy for detecting cardiac ATTR amyloid in a large population of patients with endomyocardial biopsy-proven cardiac amyloid, the current diagnostic gold-standard.

**Methods**

All patients with endomyocardial biopsy-proven cardiac amyloid who underwent 99mTc-DPD scintigraphy were included in the analysis. Delayed whole body scan images were graded as Perugini 0-3. Planar quantitation was performed on all scans using both a heart to contralateral (H/CL), and heart retention to whole body region of interest (HR/WBR) ratio, as previously reported.

**Results**

Two hundred sixty-two patients were included in the analysis, 201 with ATTR (wild-type in 136), 55 with AL, four with AApopA4 and two with AApoA1 cardiac amyloid. 99mTc-DPD scans were positive in 200/201 patients with ATTR and 33/55 (60%) patients with AL amyloid (21 grade 1 uptake, eight grade 2 and four with grade 3). Both patients with AApoA1 amyloid had a grade 1 scan and none of those with AApoA4 amyloid demonstrated cardiac tracer uptake. A positive (Perugini grade 1-3) 99mTc-DPD scan on its own was 99.5% sensitive but only 43% specific for diagnosing cardiac ATTR amyloid. A 99mTc-DPD scan with ≥ grade 2 uptake was 96% sensitive and 80% specific for ATTR amyloid, similar to that reported for pyrophosphate (PYP) scintigraphy by Bokhari and colleagues (97% sensitivity, 83% specificity), whilst Cappelli et al reported 93% sensitivity and 100% specificity using hydroxymethylene diphosphonate (HMDP). ROC analyses of H/CL and HR/WBR methods of planar quantitation demonstrated an AUC for both of 0.962 (*p*<0.001) for differentiating cardiac ATTR from non-ATTR using 99mTc-DPD. A H/CL ratio cut-off of 2.06 was 91% sensitive and 93% specific whilst a HR/WBR ratio cut-off of 3.07 yielded 92% diagnostic sensitivity and specificity for cardiac ATTR amyloid.

**Conclusions**

Bone scintigraphy with DPD, like PYP and HMDP, is extremely sensitive for diagnosing cardiac ATTR amyloid. Although the visual score (Perugini grade) and planar quantitation can lead to improved specificity, they should not be relied upon, in isolation, to differentiate between ATTR and non-ATTR forms of cardiac amyloidosis.

#### P26 Spectrum and significance of CMR findings in cardiac transthyretin amyloidosis

##### Ana Martinez-Naharro^1^, Thomas Treibel^2^, Amna Abdel-Gadir^2^, Heerajnarain Bulluck^2^, Giulia Zumbo^1^, Daniel Knight^1^, Tushar Kotecha^1^, Rohin Francis^1^, David Hutt^1^, Tamer Rezk^1^, Stefania Rosmini^1^, Cristina Quarta^1^, Carol Whelan^1^, Peter Kellman^3^, Julian Gillmore^1^, Philip Hawkins^1^, Marianna Fontana^1^

###### ^1^National Amyloidosis Centre, UCL, London, UK; ^2^Barts Heart Centre, West Smithfield, London, UK; ^3^National Institutes of Health/NHLBI, Laboratory of Cardiac Energetics, Bethesda, MD, USA

####### **Correspondence:** Ana Martinez-Naharro (a.naharro@ucl.ac.uk)

**Background**

Cardiac transthyretin amyloidosis (ATTR amyloidosis) is an increasingly recognised cause of heart failure. Cardiovascular magnetic resonance (CMR) with late gadolinium enhancement (LGE) and T1 mapping is emerging as a reference standard for diagnosis and characterisation of cardiac amyloid.

**Materials and methods**

We used CMR with extracellular volume fraction (ECV) measurement to characterise cardiac involvement in relation to outcome in ATTR amyloidosis. Subjects comprised 263 patients with cardiac ATTR amyloidosis corroborated by grade 2-3 99mTc-DPD cardiac uptake, 17 with suspected cardiac ATTR amyloidosis (grade 1 99mTc-DPD) and 12 asymptomatic individuals with amyloidogenic transthyretin (TTR) mutations. Fifty patients with cardiac AL amyloidosis acted as disease controls.

**Results**

In contrast to AL amyloidosis, asymmetric septal hypertrophy was present in 79% of ATTR patients (70% sigmoid septum and 30% reverse septal curvature), whilst symmetric left ventricular hypertrophy (LVH) was present in only 18%; 3% of patients has no LVH. In patients with cardiac amyloidosis, the pattern of LGE was always typical for amyloidosis (29% subendocardial, 71% transmural) including right ventricular LGE (96%). 65 patients died during follow-up (19±14months). ECV independently correlated with mortality and remained independent after adjustment for age, N-terminal pro-brain natriuretic peptide, ejection fraction, E/E’ and left ventricular mass index (hazard ratio, 1.164; 95% confidence interval, 1.066-1.271; *P*<0.01).

**Conclusions**

Asymmetric hypertrophy, traditionally associated with hypertrophic cardiomyopathy, is the commonest pattern of ventricular remodelling in ATTR amyloidosis. LGE imaging is typical in all patients with cardiac ATTR amyloidosis. ECV correlates with amyloid burden and provides incremental information on outcome even after adjustment for known prognostic factors.

#### P27 Clinical utility of T1 mapping in cardiac ATTR amyloidosis – diagnostic performance and prognostic capability

##### Ana Martinez-Naharro, Karl Norrington, Andrea Baggiano, Tushar Kotecha, Rohin Francis, Tamer Rezk, Cristina Quarta, Esther Gonzalez, Carol Whelan, Helen Lachman, Daniel Knight, Ashutosh Wechalekar, Julian Gillmore, Philip Hawkins, Marianna Fontana

###### National Amyloidosis Centre, UCL, London, UK

####### **Correspondence:** Ana Martinez-Naharro (a.naharro@ucl.ac.uk)

**Background**

Heart failure caused by transthyretin amyloidosis (ATTR) is underdiagnosed and has an overlapping clinical phenotype with hypertrophic cardiomyopathy (HCM). Native myocardial T1 mapping by CMR is useful for diagnosis in cardiac amyloidosis. We investigated the diagnostic and prognostic value of T1 mapping in the largest ATTR population studied so far as well as patients with HCM.

**Materials and methods**

We aimed to assess the ability of native T1 to 1) diagnose cardiac amyloidosis and 2) stratify prognosis. 134 wild-type ATTR (ATTRwt) (122 males, age 76± 7 years), 81 mutant-type (ATTRm) (60 males, age 69 ± 11 years) and 12 mutation carriers (4 males, age 47 ± 10 years) were compared to 44 HCM patients. All subjects underwent CMR with standard SSFP-cine imaging and T1 mapping. ATTR patients underwent Tc-DPD scintigraphy, the current diagnostic imaging reference standard for ATTR.

**Results**

Native T1 was elevated in ATTR compared to HCM (*p*<0.001) (mean T1: in ATTRwt 1092 ± 51ms, in ATTRm 1086 ± 67ms, in HCM 1026 ± 64ms). No significant difference between native T1 was found between ATTRwt and ATTRm. Native T1 diagnostic performance was similar for ATTRwt and ATTRm (AUC 0.865). Native T1 tracked amyloid burden (*p* < 0.001) measured by DPD scintigrapgy. During follow-up, 95 deaths occurred: 55 ATTRwt, 40 ATTRm. Native T1 was predictive of death (HR 1.225; 95% confidence interval, 1.010-1.486; *p*<0.05).

**Conclusion**s

CMR-determined native myocardial T1 has excellent diagnostic accuracy for identification of ATTR cardiac amyloidosis, tracks DPD-determined amyloid burden well and correlates with prognosis.

#### P28 The effect of tracer kinetics on heart to contralateral ratio in 99mTc-DPD scintigraphy

##### Joanne Page^1^, David Hutt^1^, Maria Burniston^2^, Julian Gillmore^1^, Philip Hawkins^1^

###### ^1^National Amyloidosis Centre, London, UK; ^2^Barts Health NHS Trust, London, UK

####### **Correspondence:** Joanne Page (joanne.page1@nhs.net)

**Background**

99mTechnetium labelled 3,3-diphosphono-1,2-propanodicarboxylic acid (99mTc-DPD) is a bone tracer used for imaging cardiac amyloid deposits. However the kinetics of its uptake and clearance in amyloid deposits and other body tissues is not well understood, and therefore it is unclear how quantitative measures would be affected by the choice of measurement time point, given that different imaging protocols have developed at various centres.

A commonly used measure is the heart to contralateral ratio (H/CL) on planar imaging. This measure aims to cancel out the effect of overlying bone and soft tissue in the heart region by mirroring the region of interest (ROI) across the body and it might be hypothesised that it would be minimally sensitive to changes in measurement time points. This work seeks to test this hypothesis.

**Materials and methods**

Nineteen patients under investigation for cardiac amyloidosis underwent static imaging over the thorax at multiple timepoints up to 4 hours post-injection in addition to their routine whole body scan at 3 hours. H/CL was calculated for each timepoint and the range in H/CL between 1 and 4 hours post-injection was calculated for each patient.

**Results**

Patients were grouped by Perugini grade and an average patient range in H/CL was calculated for each group: grade 0 = 0.21 (*n*=4); grade 1 = 0.23 (*n*=6); grade 2 = 0.45 (*n*=5); grade 3 = 0.14 (*n*=4). In the grade 0, 1 and 3 patients there was a slight downward trend in H/CL over time, mirroring a trend noted in PYP patients by Castane et al [Castano, A et. al. Multicenter Study of Planar Technetium 99m Pyrophosphate Cardiac Imaging Predicting Survival for Patients With ATTR Cardiac Amyloidosis. JAMA Cardiol. 2016;1(8):880-889.] However an upward trend with a greater range in H/CL was seen in grade 2 patients.

**Conclusions**

While uptake of 99mTc-DPD in different tissues has previously been demonstrated to be dependent on the timepoint at which it is measured, H/CL seems to remain relatively constant between 1 and 4 hours post-injection, although this variation can be significant for grade 2 patients. These differences need to be considered when combining results from different centres.

#### P29 The relationship between 99mTc-DPD uptake and amyloid fibril composition in hereditary cardiac TTR amyloidosis: is the Glu92Lys variant an exception to the rule?

##### Laura Obici^1^, Stefano Perlini^2^, Roberta Mussinelli^1^, Eloisa Arbustini^3^, Masayoshi Tasaki^4^, Francesca Lavatelli^1^, Simona Casarini^1^, Ambra Raimondi^5^, Giampaolo Merlini^6^

###### ^1^Amyloidosis Research and Treatment Centre, Fondazione IRCCS Policlinico San Matteo, Pavia, Italy; ^2^Clinica Medica II, Fondazione IRCCS Policlinico San Matteo and Department of Internal Medicine, University of Pavia, Pavia, Italy; ^3^Centre for Inherited Cardiovascular Diseases, Fondazione IRCCS Policlinico San Matteo, Pavia, Italy; ^4^Amyloidosis Research and Treatment Centre, Fondazione IRCCS Policlinico San Matteo, Department of Neurology and Department of Morphological and Physiological Sciences, Kumamoto University, Kumamoto, Japan; ^5^Clinica Medica II, Fondazione IRCCS Policlinico San Matteo, Pavia, Italy ^6^Amyloidosis Research and Treatment Centre, Fondazione IRCCS Policlinico San Matteo and Department of Molecular Medicine, University of Pavia, Pavia, Italy

####### **Correspondence:** Laura Obici (l.obici@smatteo.pv.it)

**Background**

99mTc-DPD scintigraphy identifies cardiac transthyretin (TTR) amyloid with high sensitivity and specificity (Gillmore et al. Circulation 2016). This method, however, fails to detect amyloid cardiomyopathy in a subset of patients with Val30Met amyloidosis. It has been proposed (Pilebro et al. Upsala J Med Sci 2016) that 99mTc-DPD uptake strongly depends on the biochemical characteristics of TTR amyloid fibrils, being associated with the presence of C-terminal TTR fragments (type A fibrils).

**Case report**

A 61-year-old woman with a family history of TTR amyloidosis associated with the Glu92Lys variant was referred for suspected amyloid heart involvement. Her past medical history was uneventful, except for bilateral carpal tunnel syndrome surgically treated 11 and 9 years before, respectively. Echocardiographic findings were consistent with amyloid infiltration, including increased wall thickness (IVS and PW 15 mm) with preserved ejection fraction (68%) and a restrictive filling pattern (E/A 2,9). A neurological examination was negative, with a normal nerve conduction study. NT-proBNP was 1940 pg/ml (u.r.l. < 334), TnI 0,044 ng/ml (u.r.l. < 0,040). DNA sequencing confirmed the presence of the p.Glu92Lys variant. An abdominal fat aspirate was negative. Unexpectedly, 99mTc-DPD scintigraphy didn't show uptake of the tracer in the heart (grade 0). To clarify the diagnosis and enrol the patient in a clinical trial, an endomyocardial biopsy was performed that showed the presence of amyloid deposits stained by anti-TTR antibody. Amyloid proteins were extracted from frozen cardiac tissue and fibril composition was evaluated by protein separation on 16% acrylamide gel (tris-tricine system) and immunoblotting with antiserum against TTR 50-127 (kindly provided by Prof. P. Westermark). Western blot showed abundant full-length and fragmented TTR in similar amounts, characterizing the fibrils as type A.

**Conclusion**

The sensitivity of 99mTc-DPD scintigraphy in detecting cardiac ATTR deposits may vary according to the amount of amyloid infiltration, the composition of TTR fibrils and, possibly, additional factors related to the specific amino acid substitution. While the mechanisms underlying the affinity of bone tracers to TTR amyloid remain undetermined, lack of DPD uptake in the presence of echocardiographic findings consistent with amyloidosis should not dismiss the diagnostic suspicion and should prompt genetic testing and/or biopsy of the involved organ.

**Consent to publish**

Written informed consent was obtained from the patients involved in this study.

#### P30 Comparison of immunohistochemistry and proteomic analysis for identification and typing of amyloid in different histological tissues

##### Janet A. Gilbertson, Tamer Rezk, Nigel B. Rendell, Graham W. Taylor, Patrizia Mangione, Diana Canetti, Vittorio Bellotti, Philip Hawkins, Julian Gillmore

###### National Amyloidosis Centre, Division of Medicine, Royal Free Campus, UCL Medical School, London, UK

####### **Correspondence:** Janet A. Gilbertson (j.gilbertson@ucl.ac.uk)

**Background**:

Accurate identification of the amyloid fibril protein is essential. Immunohistochemistry (IHC) fails to type amyloid in up to 30% cases. Proteomic analysis of amyloidotic material by mass spectrometry is reported to be a powerful tool for identifying the amyloid fibril protein in fixed tissue sections. We report proteomic findings in patients referred to the UK National Amyloidosis Centre.

**Materials and methods**:

Two hundred seventy-one biopsies from 30 different tissue types were evaluated by Congo red (CR) staining, IHC and proteomic analysis. Proteomic findings and IHC were interpreted by two experienced operators, blind to any clinical details.

**Results**:

Of the 272 biopsies, 251 contained amyloid (CR+) and 21 did not (CR-). Presence of amyloid was supported by at least 2 amyloid signature proteins (SP: SAP, apoA-IV or apoE), in the proteome of 225/251 (90%) CR+ biopsies (181/251 contained all 3 SPs, 44/251 had 2 of 3 SPs. The ‘amyloid signature’ was not identified in the proteome of 26/251 (10%) CR+ samples, with 12 (4%) containing only one SP and 14 (6%) no SPs. Absence of the ‘amyloid signature’ supported CR- staining in 20/21 (95%) samples, but the proteome of 1/21 CR- case revealed AL (kappa sub-type) amyloid on the basis of presence of all 3 SPs and kappa light chain. In 125/251 (50%) CR+ samples, the amyloid subtype was definitively established by IHC, and supported in 114/125 (91%) cases by proteomic analysis. In 11/125 (9%) samples, results of IHC and proteomics differed for the following reasons: no amyloid SPs by proteomics (*n*=2/11), uncertain fibril protein by proteomics (*n*=8/11), different fibril protein (lambda by IHC, kappa by proteomics) (*n*=1/11). Of 126/251 (50%) CR+ samples with amyloid of indeterminate type by IHC, the amyloid sub-type was determined in 103/126 (82%) cases. The remaining 23 cases were not definitively typed by either IHC or proteomic analysis, 7/126 (6%) containing no SPs by proteomics, and 16/126 (12%) yielding uncertain results by proteomics.

**Conclusions**:

Proteomic analysis revealed false negative and false positive results for amyloid in 10% and 5% of cases respectively. IHC concurred with proteomic analyses in determining the amyloid subtype in 91% of cases. The amyloid subtype was successfully determined in over 80% of samples in which IHC was inconclusive. Proteomic analysis and IHC are complimentary techniques for diagnosis and typing of amyloid and should be interpreted in the context of the overall clinical picture.

#### P32 A novel serum microrna signature to screen ATTR

##### M'hammed Aguennouz^1^, Anna Mazzeo^1^, Claudia Stancanelli^1,2^, Francesca Polito^1^, Marco Ragusa^3^, Roberto Arrigo^1^, Luca Gentile^1^, Anna Maria Ciranni^1^, Rosa Maria Di Giorgio^1^, Giuseppe Vita^1^

###### ^1^Department of Clinical and Experimental Medicine, University of Messina, Messina, Italy; ^2^Biomedical Department of Internal Medicine and Specialistic, University of Palermo, Palermo, Italy; ^3^Department BIOMORF, University of Catania, Catania, Italy

####### **Correspondence:** M'hammed Aguennouz (aguenoz@unime.it)

**Background**

Transthyretin amyloidosis (ATTR), is the most common form of genetic amyloidosis. It is a progressive devastating disease presenting with a heterogeneous spectrum of manifestations, thus diagnosis is often overlooked. Given that ATTR is now susceptible of treatment and early diagnosis provides for better prognosis, potential biomarkers are needed. Circulating microRNAs (miRNAs) have been described as promising diagnostic biomarkers in many chronic and degenerative neurological disorders. miRNAs show an advantage as biomarkers, being exceptionally stable among blood macromolecules, as they have been reported to be transported in blood on exosomes, high-density lipoproteins, and on complexes with proteins, protecting them from degradation. To evaluate the expression of miRNAs in sera from symptomatic and asymptomatic ATTR patients from South Italy, carrying three endemic mutations, and to identify a miRNA cluster as potential biomarker predictive of disease’s progression.

**Materials and methods:**

A quantitative reverse transcription polymerase chain reaction (qRT-PCR) of an array panel containing approximately 836 human miRNAs.

**Results and conclusions:**

Bioinformatics tools showed an involvement of autophagy and apoptotic pathways in ATTR patients vs asymptomatic and pathological controls (other genetic neuropathies), suggesting to use miRNAs as potential biomarkers of disease.

#### P33 Strange location for diagnosis of ATTR amyloidosis

##### Manuel Raya-Cruz^1^, Juan Buades-Reinés^1^, Cristina Gallego-Lezaún^1^, Tomás Ripoll-Vera^2^, Mercedes Usón-Martín^3^, Eugenia Cisneros-Barroso^3^

###### ^1^Department of Internal Medicine, Son Llàtzer Hospital, Palma De Mallorca, Spain; ^2^Department of Cardiology, Son Llàtzer Hospital, Palma De Mallorca, Spain; ^3^Department of Neurology, Son Llàtzer Hospital, Palma De Mallorca, Spain

####### **Correspondence:** Manuel Raya-Cruz (manuelraya@hotmail.com)

**Background**

ATTR amyloidosis is an entity characterized by distal sensorimotor polyneuropathy, autonomic disturbances, autosomal dominant heredity. Val30Met is the most common mutation ATTR patients. Histological diagnosis consists of prospective biopsy of nerves or other tissues like skin, subcutaneous abdominal fat, rectal mucosa and labial salivary glands. To describe a rare case where the presence of amyloid in the nasal mucosa is demonstrated in a Val30Met familial amyloid polyneuropathy (FAP) patient.

**Case report**

A 60-year-old woman, also a carrier of the TTRV30M mutation. She has loss of tactile sensation and pain in her legs. Electromyography showed a chronic axonal polyneuropathy, for which an abdominal fat biopsy was performed where it was, evidenced positive Congo red staining and polarization microscopy revealed amyloid deposition. Liver transplantation was performed, outcome was favorable but later presented clinical worsening with progressively increased pain and loss of sensitivity of legs and arms. Four years later she was seen for a history of obstruction and epistaxis of the right side of her nose. An anterior rhinoscopy showed a delimited and vascularised mass occupying nasal fossa. A nasal biopsy was performed, Congo red staining and polarization microscopy revealed chronic non-specific inflammation of the nasal mucosa with small amyloid deposits in deep vessels. The specimen consisted of several polypoid fragments of nasal mucosa, with focal inflammatory infiltrate and ulceration, the base of the polyp contained several thick-walled vessels, some of which showed focal amyloid deposits but not in newly formed vessels either in stroma of the polyp, thus excluding a new onset amyloid deposition. This was not an amyloid tumour, but a non-specific inflammatory polyp arising on mucosa containing vessels previously involved by amyloid. We cannot rule out that the clinical worsening of the patient at the neurological level is a reflection of the presence of amyloid material in the nasal mucosa, although the deposition in the present case had previously been larger but had diminished after liver transplant.

**Conclusion**

We report the case of a female patient with ATTR amyloid deposition in the inferior nasal conchal vessels, was performed incidentally by a nonspecific inflammatory polyp. Small focal deposits of amyloid TTR were observed on deep thick walled vessels. This location could be a suitable biopsy site.

**Consent to publish**

Written informed consent was obtained from the patients involved in this study.

#### P34 Peripheral nerve ultrasonography in patients with transthyretin amyloidosis

##### Zidar, J.^1^, Sarafov, S.^2^, Tournev, I.^2,3^, Omejec, G.^1^, Podnar, S.^1^

###### ^1^Institute of Clinical Neurophysiology, Division of Neurology, University Medical Centre Ljubljana, Slovenia; ^2^Department of Neurology, Alexandrovska University Hospital, Sofia Medical University, Sofia, Bulgaria; ^3^Department of Cognitive Science and Psychology, New Bulgarian University, Sofia, Bulgaria

####### **Correspondence:** Zidar, J. (janez.zidar@kclj.si)

**Background**

We aimed to systematically study peripheral nerve morphology in patients with transthyretin (TTR) amyloidosis and TTR gene mutation carriers using high-resolution ultrasonography (US).

**Materials and methods**

In this prospective cross-sectional study we took a structured history, performed neurological examination, and measured peripheral nerve cross-sectional areas (CSAs) bilaterally at 28 standard locations using US. Demographic and US findings were compared to controls.

**Results**

Peripheral nerve CSAs were significantly larger in 33 patients with familial amyloid polyneuropathy (FAP) compared to 50 controls, most dramatically at the common entrapment sites (median nerve at the wrist, ulnar nerve at the elbow), and in the proximal nerve segments (median nerve in the upper arm, sciatic nerve in the thigh). Findings in 21 asymptomatic TTR gene mutation carriers were less marked compared to controls, with CSAs being larger only in the median nerve in the upper arm. Nerve CSAs correlated with abnormalities on nerve conduction studies.

**Conclusions**

Using US, we confirmed previous pathohistological and imaging reports in FAP of the most pronounced peripheral nerve thickening in the proximal limb segments. Similar to US findings in diabetic and vasculitic neuropathies these predominantly proximal locations of nerve thickening may be attributed to ischaemic nerve damage caused by poor perfusion in the watershed zones along proximal limb segments.

### Topic: Update in the Treatment for ATTR Amyloidosis

#### P37 Tafamidis delays disease progression comparably across Val30Met and non-Val30Met genotypes in transthyretin familial amyloid polyneuropathy

##### Balarama Gundapaneni^1^, Marla Sultan^2^, Jeffrey Schwartz^2^, Denis Keohane^2^

###### ^1^inVentiv Health Inc. Burlington, MA, USA; ^2^Pfizer, New York, NY, USA

####### **Correspondence:** Balarama Gundapaneni (balarama.gundpaneni@pfizer.com)

**Background**

Patients with transthyretin (TTR) familial amyloid polyneuropathy (TTR-FAP) can be broadly divided into two groups based on their TTR genotype: those carrying a Val30Met mutation and those carrying a non-Val30Met mutation. Recent evidence suggests that the overall prognosis of Val30Met patients is better than that of non-Val30Met patients.

**Materials and methods**

We therefore conducted a post hoc analysis comparing data from Val30Met patients who participated in the 18-month registration trial of tafamidis, a selective kinetic stabilizer of TTR shown to delay neurologic progression and the only medicine approved for the treatment of TTR-FAP, with data from a 12-month, open-label trial of tafamidis that included non-Val30Met patients. The data was comprised of three groups: tafamidis-treated Val30Met (*n*=64); tafamidis-treated non-Val30Met (*n*=21); and placebo-treated Val30Met (*n*=61). The severity and progression of neurologic impairment was evaluated using the Neuropathy Impairment Score-Lower Limbs (NIS-LL) scale, and a mixed-effects model for repeated measures (MMRM) was used to compare the efficacy of tafamidis versus placebo.

**Results**

The baseline characteristics of the three groups showed that non-Val30Met patients were older, had a longer duration of symptoms, and had a higher level of baseline neurologic impairment compared with the Val30Met patients. Following 12 months of treatment, baseline-adjusted changes in NIS-LL were similar in the 2 tafamidis-treated groups (mean ± standard error: 1.60 ± 0.78 and 1.62 ± 1.43 in the Val30Met and non-Val30Met groups, respectively), but was greater in the Val30Met placebo-treated group (4.72 ± 0.77; *p*=0.0055 and *p*=0.0592 versus the Val30Met and non-Val30Met tafamidis-treated groups, respectively). The MMRM analysis predicted similar changes in NIS-LL for the Val30Met and non-Val30Met tafamidis-treated groups across a range of baseline NIS-LL scores, and the predicted changes for both these groups were consistently smaller than those predicted for the placebo-treated group. The MMRM also predicted that the extent of disease progression increased as baseline NIS-LL increased.

**Conclusions**

This post hoc MMRM analysis demonstrated that tafamidis delayed neurologic progression of TTR-FAP to a similar extent in patients with a Val30Met or non-Val30Met TTR mutation. Sponsored by Pfizer Inc. ClinicalTrials.gov identifiers: NCT00409175; NCT00630864.

#### P39 Influence of baseline neurologic severity on disease progression and the associated disease-modifying effects of tafamidis in transthyretin familial amyloid polyneuropathy

##### Leslie Amass^1^, Huihua Li^1^, Balarama Gundapaneni^2^, Jeffrey Schwartz^1^, Denis Keohane^1^

###### ^1^Pfizer, New York, NY, USA; ^2^inVentiv Health Inc. Burlington, MA, USA

####### **Correspondence:** Leslie Amass (leslie.amass@pfizer.com)

**Background**

A number of factors can influence disease progression in transthyretin familial amyloid polyneuropathy (TTR-FAP), a rare, progressive, and fatal hereditary amyloidosis.

**Materials and methods**

This analysis utilized longitudinal data from 5.5 years of exposure in the clinical trials of tafamidis, a selective TTR stabilizer, to evaluate the impact of baseline neurologic severity on disease progression in TTR-FAP. A linear mixed-effects model was constructed using data from the intent-to-treat Val30Met patients in the tafamidis registration trial and its 2 consecutive open-label extensions. The second extension is ongoing but an interim analysis involving a cleaned and locked database has been conducted (cut-off: December 31, 2014). Groups in the current analysis consisted of patients who received tafamidis during the registration and open-label studies (T-T group), or who received placebo during the registration trial and tafamidis during the open-label extensions (P-T group). This analysis focused on the first 18 months of treatment and disease progression as measured by the Neuropathy Impairment Score-Lower Limbs (NIS-LL).

**Results**

The T-T (*n*=64) and P-T (*n*=61) groups included approximately equal numbers of men and women (mean age: 39 years) with early stage neurologic disease at baseline (mean NIS-LL ± standard deviation: 8.4 ± 11.4 and 11.4 ± 13.5, respectively). The slopes (rates of change) for NIS-LL from baseline to Month 18 were different across baseline NIS-LL scores (*p*<0.0001): patients with a lower baseline NIS-LL showed less progression than those with a higher baseline NIS-LL. Neurologic progression in the T-T group was less than in the P-T group across all levels of baseline NIS-LL (*p*=0.0004), and the degree of separation increased with longer durations of treatment. Similar results were seen in the NIS-LL muscle weakness subscale. These findings illustrate that neurologic progression depends strongly on baseline neurologic severity. The disease-modifying effect of tafamidis treatment relative to placebo was seen across the range of baseline levels of neurologic severity and treatment durations. The differences in the slopes of disease progression across groups support an increasing clinical benefit from tafamidis treatment over time.

**Conclusions**

Overall, this analysis supports the value of tafamidis treatment in patients with TTR-FAP. Sponsored by Pfizer Inc. ClinicalTrials.gov identifiers: NCT00409175, NCT00791492, NCT00925002.

#### P40 Reasons for discontinuation of TTR stabilizers and/or TTR fibril disrupter: an analysis of baseline demographics of patients with hereditary ATTR (hATTR) amyloidosis in the phase 3 apollo study

##### Angela Partisano^1^, Hartmut Schmidt^2^, William O’riordan^3^, Taro Yamashita^4^, John L. Berk^5^, Ivaylo Tournev^6^, Michelle Mezei^7^, Violaine Plante-Bordeneuve^8^, Yoshiki Sekijima^9^, Ole Suhr^10^, Giuseppe Vita^11^, Jihong Chen^1^, Jared Gollob^1^, David Adams^12^

###### ^1^Alnylam Pharmaceuticals, Cambridge, MA USA; ^2^University Hospital of Muenster, Muenster, Germany; ^3^eStudy Site, La Mesa, CA, USA; ^4^Kumamoto University Hospital, Kumamoto, Japan; ^5^Amyloidosis Center, Boston University, Boston, MA, USA; ^6^University Multiprofile Hospital for Active Treatment, Sofia, Bulgaria; ^7^Vancouver General Hospital, Vancouver, Canada; ^8^CHU Henri Mondor, Creteil, France; ^9^Shinshu University Hospital, Nagano, Japan; ^10^Umeå University Hospital, Umeå, Sweden; ^11^Policlinico Universitario G. Martino, Sicily, Italy; ^12^National Reference Center for FAP, CHU Bicêtre, APHP, Le Kremlin-Bicêtre, France

####### **Correspondence:** Angela Partisano (apartisano@alnylam.com)

**Background**

hATTR amyloidosis is a rapidly progressive, life-threatening disease. Currently, only tafamidis is approved for hATTR amyloidosis and approval is limited to select countries outside the US. Recent data suggest disease progression may occur with tafamidis (Planté-Bordeneuve. J Neurol 2017). Diflunisal (NSAID) shown to prevent dissociation of TTR tetramers, and doxycycline (TTR fibril disrupter) are sometimes used off-label for hATTR amyloidosis. Patisiran, an investigational RNAi therapeutic targeting hepatic TTR production, is being studied in a Phase 3 trial (APOLLO). This abstracts describe the demographics of patients receiving tafamidis, diflunisal, and/or doxycycline prior to APOLLO enrollment and physician-reported reasons for discontinuation.

**Materials and methods**

APOLLO is a Phase 3 multi-center, international, randomized, double-blind, placebo-controlled study (NCT01960348) to evaluate the efficacy and safety of patisiran in patients with hATTR amyloidosis with polyneuropathy. Symptomatic patients with documented TTR mutation and NIS 5-130 were eligible. Select exclusion criteria: prior liver transplantation, PND >IIIb, NYHA Class >2. Patients receiving tafamidis/doxycycline or diflunisal were required to discontinue ≥14 or > 3 days before patisiran administration, respectively. Primary endpoint is change from baseline at 18 mos in mNIS+7 composite neurologic impairment score.

**Results**

APOLLO enrolled 225 patients with hATTR amyloidosis with polyneuropathy. Among these, 119 patients were previously treated with tafamidis (62%), diflunisal (33%), or diflunisal/doxycycline combination (5%). Mean age: 61 yrs (range 27-83); males: 76%, V30M: 45%; non-V30M: 55%. Disease severity measures, mean NIS: 58 points (6-142); mean KPS: 71 (60-100); PND I: 23%; PND II: 34%; PND IIIa: 29%; PND IIIb: 15%; NYHA Class 1, 2: 50% each. Physician-reported reasons captured at time of enrollment for patient discontinuation of therapies prior to APOLLO enrollment: clinical study enrollment (72%), disease progression (20%), safety (1%), or other (7%).

**Conclusions**

APOLLO, the largest, controlled study of patients with hATTR amyloidosis with polyneuropathy, includes patients with a wide range of TTR genotypes and neuropathy severity. Of patients previously treated with TTR stabilizers/disrupters, >90% discontinued treatment for APOLLO eligibility or disease progression on therapy. Data highlight significant unmet need in patients with hATTR amyloidosis with polyneuropathy.

#### P41 Characteristics of patients with hereditary transthyretin-mediated amyloidosis on the US liver transplant waiting list

##### Sonalee Agarwal^1^, Danielle Brandman^2^, Saša Živkovic^3^, William Irish^4^, Larry Gache^4^, Anastasia Mcmanus^1^, Jared Gollob^1^, Angela Partisano^1^

###### ^1^Alnylam Pharmaceuticals, Cambridge, MA, USA; ^2^Hepatology and Liver Transplantation, University of California, San Francisco, CA, USA; ^3^Department of Neurology, University of Pittsburgh, Pittsburgh, PA, USA; ^4^CTI Clinical Trial & Consulting Services, Cincinnati, OH, USA

####### **Correspondence:** Sonalee Agarwal (sagarwal@alnylam.com)

**Background**

Hereditary transthyretin-mediated amyloidosis (hATTR amyloidosis), formerly known as familial amyloidosis, is an inherited, rapidly progressive, life-threatening, multisystemic disease leading to mortality in 2-15 years. Orthotopic liver transplantation (OLT) is an option for patients with early-onset hATTR amyloidosis and short disease duration. Understanding the outcomes of patients during the waiting period is important given the rapid course of disease progression. This study aims to describe demographic and clinical characteristics of patients with hATTR amyloidosis on the liver transplant waiting list and transplanted list using the OPTN/UNOS liver transplant population-based registry.

**Materials and methods**

Adult patients registered for the first time on the OPTN/UNOS liver transplant waiting list with hATTR amyloidosis (exception diagnosis code of familial amyloidosis) between March 2002 - March 2016 were identified and followed through December 2016. Demographics and clinical outcomes were collected.

**Results**

A total of 225 patients with hATTR amyloidosis were listed for OLT as of March 2016. Characteristics and clinical outcomes of patients are summarized in Table 1. Among the patients listed, 170 (76%) underwent OLT, 18 (8%) did not receive treatment because they were too sick or died prior to organ availability, 21 (9%) withdrew, and 16 (7%) were still waiting as of December 2016. The average time between wait list entry and removal for any reason was 286 days. Of the patients who received OLT, 12% of patients had a functional status ≤50% at initial listing which rose to 23% of patients with a functional status of ≤50% at time of OLT, suggestive of disease progression while awaiting transplant. While majority of patients with hATTR amyloidosis on the OPTN/UNOS transplant list received an OLT in less than 1 year, the functional status of patients deteriorated.

**Conclusions**

These data highlight the significant unmet need in patients with hATTR amyloidosis. If new therapeutic options emerge, there may be more options for patients to manage their disease and potentially provide an alternative to OLT.

**Table 1 (abstract P41).** Characteristics of Wait List and Liver Transplant Cohort

	Cohort at listing time (*n*=225)	Cohort at time of removal for OLT (*n*=170)
Characteristics & Clinical Outcomes		
Mean age (SD) years	53.4 (10.7)	53.6 (10.7)
Males	73.8%	72.4%
Mean mBMI (SD) kg/m^2^ x g/L	1032.3 (239.2)	1007.1 (261.3)
Mean SCr (SD) mg/dL	1.3 (1.4)	1.0 (0.4)
Medical condition between listing and OLT		
ICU	-	6.5%
Hospitalized not in ICU	-	3.5%
Not hospitalized	-	90%

#### P42 Cardiac involvement after liver transplantation in patients with Val30Met transthyretin amyloidosis from Majorca focus

##### Tomas Ripoll-Vera, Juan Buades, Eugenia Cisneros, Yolanda Gomez, Juana Nuñez, Manuel Raya, Cristina Gallego, Maria Asuncion Ferrer, Mercedes Uson, Hernan Andreu, Antonio Figuerola, Jorge Alvarez

###### Hospital Son Llatzer & Idisba, Palma De Mallorca, Spain

####### **Correspondence:** Tomas Ripoll-Vera (tripoll@hsll.es)

**Background**

Familial amyloid polyneuropathy (FAP) is an autosomal dominant disease caused by mutations in transthyretin (TTR) gene. Val30Met is the most common variant. Although rare worldwide, there are descriptions of some endemic foci, such as in Majorca (Spain). The TTR Val30Met mutation presents classically with peripheral sensory neuropathy and progresses to autonomic and motor neuropathy, with occurrence of cardiac conduction abnormalities late in the disease progression. Orthotopic liver transplantation (OLT) is considered the best treatment. The abnormal TTR protein is synthesized in 95% in the liver, but also in retina and choroid plexus. Thus, it could be some worsening in spite of OLT. There are few published data about cardiac disease in post-OLT FAP patients.

The aim of this study was to investigate the occurrence and development of heart symptoms, arrhythmias, conduction abnormalities, and myocardial involvement in Majorca TTR Val30Met FAP patients who underwent OLT.

**Materials and methods**

Retrospective observational study selecting those patients who underwent OLT, and comparing cardiac involvement prior to OLT and after follow up.

**Results**

The cohort comprised 132 FAP patients (69 males and 63 females). 54(41%) had received an OLT. Thirty-two(60%) were men. The mean age was 42.3±12.6 years at diagnosis. The time to inclusion on the transplant waiting list was 29.56 months. During follow up after OLT (14.4 years) several patients showed disease progression referred to neuropathy, nephropathy (more related to immunosuppression drugs) and cardiopathy (26.4% preOLT vs 61.5% postOLT), specially rhythm disturbances (9 vs 38.5%), atrium-ventricular block (AVB) (9 vs 35%), heart failure (HF) (2 vs 10%), increasing in left ventricular hypertrophy (LVH) (10 vs 13 mm) and diastolic dysfunction (13 vs 53%). Pacemaker were implanted in 31 patients (58.5%), but 21 (39.6%) were prophylactic pre-OLT (an ancient and controversial indication). 14 patients died (26.4%), the majority (10) FAP-related. In some cases a fatal arrhythmia was the probably cause of death. Mortality correlated with neurological worsening after OLT (*p* 0.025), nephropathy (*p* 0.008), any cardiac progression (*p* 0.04) and HF (*p* 0.014) after OLT.

**Conclusions**

There is a disease progression in FAP patients in spite of OLT, especially after long follow up, related specially to neurological and cardiac involvement. New rhythm abnormalities, AVB, HF and LVH appear after OLT, and confer a bad prognosis.

#### P43 Cardiomyopathy and peripheral polyneuropathy progression in patients with hereditary transthyretin-related amyloidosis associated with Glu89Gln mutation treated with tafamidis

##### Mariana Gospodinova^1^, Stayko Sarafov^2^, Teodora Chamova^2^, Andrey Kirov^3^, Tihomir Todorov^3^, Albena Todorova^3^, Ivailo Tournev^2^, Ivailo Tournev^4^, Stefan Denchev^1^

###### ^1^Medical Institute, Ministry of Interior, Clinic of Cardiology, Sofia, Bulgaria; ^2^University Aleksandrovska Hospital, Clinic of Neurology, Medical University, Sofia, Bulgaria; ^3^Department of Chemistry and Biochemistry, Medical University, Medicodiagnostic Lab. Genika, Sofia, Bulgaria; ^4^New Bulgarian University, Department of Cognitive Science and Psychology, Sofia, Bulgaria

####### **Correspondence:** Mariana Gospodinova (maryvg2009@yahoo.com)

**Background**

Patients with hereditary transthyretin-related amyloidosis (ATTR) with Glu89Gln mutation are characterized by mixed phenotype – cardiac and neurological. To evaluate the progression of cardiac involvement in patients with coexisting peripheral polyneuropathy, treated with Tafamidis.

**Materials and methods**

Twenty-seven patients (13 males) at mean age 57±7 years with ATTR were evaluated by clinical exam and Echocardiography. The patients have been diagnosed with Glu89Gln mutation. Tafamidis was initiated after the first assessment. The patients were followed for 23 (12-36) months on average.

**Results**

At the time of first evaluation, cardiac and neurological involvement was found in all the patients. All had signs and symptoms of peripheral polyneuropathy defined as stage 1, NYHA class 3 was found in 3 patients. At the second assessment, all patients remained in first neurological stage. A progression to NYHA class 3 was observed in 6 patients. The echocardiographic measurements revealed significant increase in left ventricular (LV) posterior wall thickness (*p*<0,02). Significant changes were found in some left ventricular (LV) diastolic function parameters (e’ septal < 0,01, e’ lateral < 0,005, a’ septal <0,03, a’ lateral <0,006, E/e’ septal < 0,002, E/e’ lateral < 0,0008, left atrium < 0,0005), but not in LV systolic function (EF, s wave, Global Longitudinal Strain – *p* > 0,05). No significant change was found in right ventricular wall thickness.

**Conclusions**

The evaluated cohort is characterized by more advanced cardiac than neurological involvement at baseline. The follow up revealed that all the patients remained in first neurological stage, but some progression in NYHA class was observed. Worsening of LV diastolic function without significant reduction in systolic function was found. Initiation of any treatment at an early stage of heart involvement may be beneficial.

#### P44 Long-term treatment of ATTR with tafamidis: the Sicilian experience

##### Luca Gentile^1^, Claudia Stancanelli^1,2^, Massimo Russo^3^, Giuseppe Vita^1^ and Anna Mazzeo^1^

###### ^1^Department of Clinical and Experimental Medicine, University of Messina, Messina, Italy; ^2^Biomedical Department of Internal Medicine and Specialistic, University of Palermo, Palermo, Italy; ^3^Nemo Sud Clinical Centre, AOU Policlinico, Messina, Italy

####### **Correspondence:** Luca Gentile (lucagentile84@yahoo.it)

**Background**

Tafamidis is a transthyretin (TTR) stabilizer able to prevent TTR tetramer dissociation. There have been a few encouraging studies on Tafamidis efficacy in early-onset inherited transthyretin amyloidosis (ATTR) due to Val30Met mutation. However, less is known about its efficacy in later disease stages and in non-Val30Met mutations.

**Materials and methods**

Here we report the experience on long term Tafamidis treatment in a ATTR population referring to our tertiary care center in Sicily, an endemic area. We studied 21 patients carrying non-Met30 mutations (Glu89Gln: n 11; Phe64Leu: n 8; Thr49Ala: 2), who had undergone Tafamidis treatment from 1 to 6 years. We evaluated modifications of BMI, NIS, FAP stage, NYHA class and CADT.

**Results**

Our results confirmed the long term well tolerability of Tafamidis, the effects on body weight preservation and the lower progression of neuropathy in a subgroup of patients.

**Conclusions**

Neuropathy and cardiomyopathy progressed in a significant proportion of patients despite treatment, mainly in subjects with poor baseline status.

#### P45 Long-term, open-label clinical experience with patisiran, an investigational RNAi therapeutic for patients with hereditary transthyretin-mediated (hATTR) amyloidosis with polyneuropathy

##### Angela M. Partisano^1^, John L. Berk^2^, David Adams^3^, Ole Suhr^4^, Isabel Conceicao^5^, Marcia Waddington Cruz^6^, Hartmut Schmidt^7^, Juan Buades^8^, Josep M. Campistol^9^, Jean-Yves Pouget^10^, Michael Polydefkis^11^, Marianne Sweetser^1^, Jihong Chen^1^, Jared Gollob^1^, Teresa Coelho^12^

###### ^1^Alnylam Pharmaceuticals, Cambridge, MA, USA; ^2^Amyloidosis Center, Boston University, Boston, MA, USA; ^3^National Reference Center for FAP, CHU Bicêtre, APHP, Le Kremlin-Bicêtre, France; ^4^Umeå University, Umeå, Sweden; ^5^Centro Hospitalar Lisboa Norte-Hospital de Santa Maria, Lisbon, Portugal; ^6^Hospital Universitário Clementino Fraga Filho, Federal University of Rio de Janeiro, Rio De Janeiro, Brazil; ^7^University of Münster, Münster, Germany; ^8^Hospital Son Llatzer, Palma De Mallorca, Spain; ^9^Hospital Clinic, University of Barcelona, Barcelona, Spain; ^10^Hôpital de La Timone, Marseille, France; ^11^Johns Hopkins University, Baltimore, MD, USA; ^12^Hospital de Santo António, Centro Hospitalar do Porto, Porto, Portugal

####### **Correspondence:** Angela M. Partisano (apartisano@alnylam.com)

**Background**

hATTR amyloidosis is a multisystemic, rapidly progressive, life-threatening disease caused by a mutation in the TTR gene, resulting in deposition of amyloid fibrils in multiple organs. Heterogeneous clinical presentation of hATTR amyloidosis includes sensory, motor and autonomic neuropathy, as well as cardiac involvement, resulting in significant morbidity and mortality. Patisiran, an investigational RNAi therapeutic targeting TTR mRNA, previously reported sustained mean reduction of serum TTR. The objective of this abstract is to describe the long-term safety and efficacy of patisiran in patients with hATTR amyloidosis with polyneuropathy.

**Materials and methods**

Patients with hATTR amyloidosis with polyneuropathy originally dosed on the Phase 2 patisiran study were eligible to roll over onto a Phase 2 OLE study and continue receiving patisiran 0.3mg/kg IV q3W for up to a total of 24 months (NCT01961921). Primary endpoint was safety; secondary objectives included: effects on neurologic impairment (mNIS+7, NIS), QoL, mBMI, mobility, grip strength, autonomic symptoms, and serum TTR levels. Following completion of the study, patients were eligible to continue treatment on a global OLE study (NCT02510261).

**Results**

Phase 2 OLE study included 27 patients; median age 64 years (range: 29-77). Patisiran given for 24 months was generally well tolerated; 7 patients experienced SAEs unrelated to study drug, including 1 patient with fatal gastroesophageal cancer. Another unrelated death (myocardial infarction) occurred after completing dosing, but prior to the final visit. Flushing (22.2%) and infusion-related reactions (22.2%) were the most common related AEs; all were mild in severity and did not result in discontinuations. Sustained mean serum TTR lowering of ~80% was achieved for >24 months (mean maximal TTR lowering: 93%) and resulted in an improvement in neuropathy with a mean 7.0-point decrease in mNIS+7 (*n*=26). A total of 25 patients subsequently enrolled onto the global OLE study.

**Conclusions**

Long-term (24 month) administration of patisiran was generally well-tolerated, resulted in robust and sustained serum TTR lowering, and supports the therapeutic hypothesis that TTR lowering can potentially halt or improve neuropathy progression in patients with hATTR amyloidosis with polyneuropathy. Safety and mNIS+7 data from patients treated for another 12 months (total of 36 months) on the global OLE study will be presented.

#### P46 Treatment of ATTR cardiomyopathy with a TTR specific antisense oligonucleotide

##### Merrill D. Benson^1^, Noel R. Dasgupta^2^

###### ^1^Department of Pathology and Laboratory Medicine, Indiana University School of Medicine, Indianapolis, IN, USA; ^2^Division of Cardiology, Indiana University School of Medicine, Indianapolis, IN, USA

####### **Correspondence:** Merrill D. Benson (mdbenson@iupui.edu)

**Background**

Cardiomyopathy is a major manifestation for patients with ATTR amyloidosis, both hereditary (hATTR) and wild-type (wtATTR). A recent phase 3 study of a TTR specific antisense oligonucleogide (Inotersen) has shown significant therapeutic effect in patients with hATTR polyneuropathy (hATTR-PN). Approximately 62% of patients enrolled in this study had concomitant hATTR cardiomyopathy (hATTR-CM), however wtATTR patients and hATTR-CM patients with insufficient or no neuropathy were not eligible.

**Materials and methods**

In a phase 2 open label study we have now treated 19 patients with ATTR cardiomyopathy (8 hATTR-CM, 11 wtATTR) with Inotersen for 12 months and of these 11 (8 hATTR-CM, 3 wtATTR) have been treated for 2 years.

**Results**

Safety profile has been good and the drug well tolerated. Cardiomyopathy parameters determined by transthoracic ECHO and MRI have remained stable in most subjects. Functional cardiac parameters (6MWT, NYHA class, LV strain, BNP) have also been consistent in most cases with lack of progression of cardiomyopathy.

**Conclusions**

In general, the results of this phase 2 study have been favorable and support pursuit of a phase 3 study in patients with ATTR cardiomyopathy.

#### P47 Decreased S100A8/A9 in V30M related familial amyloid polyneuropathy: a possible pathway in misregulation of Schwann cells

##### João Moreira^1^, Nádia P. Gonçalves^2^, Margarida Saraiva^1^, Maria João Saraiva^1^

###### ^1^I3S - Instituto de Investigação e Inovação em Saúde, Universidade do Porto, Porto, Portugal; ^2^Danish Research Institute of Translational Neuroscience DANDRITE, Nordic-EMBL Partnership,Department of Biomedicine, Aarhus University, Aarhus, Denmark

####### **Correspondence:** João Moreira (joao.moreira@ibmc.up.pt)

**Background**

Familial amyloidotic polyneuropathy (FAP) is an autosomal dominant neurodegenerative disorder with extracellular deposition of mutant transthyretin (TTR) aggregates and fibrils, particularly in nerves and ganglia of the peripheral nervous system (PNS). The most common TTR mutation is a substitution of a Methionine for a Valine at position 30 (TTR V30M) that predisposes TTR to form aggregates and fibrils. V30M human FAP nerve biopsies display increased cytokine production, but intriguingly no immune inflammatory cellular infiltrate is observed around TTR aggregates, which contributes to disease aggravation [1]. Moreover, in response to nerve injury, a V30M transgenic mice model display a downregulated innate immune response when compared to wild type (WT) mice [2]. Schwann cells are the nerve sentinel cells and have the ability to regulate the PNS immune response by secreting cytokines and chemokines thus having a central role for nerve repair. In nerves of FAP patients, Schwann cells are impaired in their ability to express chemokines, which are important drivers of tissue regeneration [3].

**Results**

In this study, we shown that the expression of S100A8/A9 molecules, known potent initiators of the immune response in stimulated Schwann cells of injured peripheral nerves, is downregulated in primary Schwann cells incubated with aggregated protein as compared to WT TTR. In line with this, we found that S100A8/A9 mRNA levels are highly decreased in V30M mice, as compared with WT controls. By ELISA, S100A8 and S100A9 protein levels were found downregulated in plasma samples from V30M FAP patients.

**Conclusions**

The presence of V30M TTR impacts S100 expression and appears to impair the immune activation of Schwann cells in V30M nerves. This may be linked to the diminished immune cellular activation and infiltration observed in FAP nerves, contributing in this way for the neuronal dysfunction present in the disease.

**References**

1. Nyhlin, N., et al., Advanced glycation end product in familial amyloidotic polyneuropathy (FAP). J Intern Med, 2000. 247(4): p. 485-92.

2. Goncalves, N.P., M. Teixeira-Coelho, and M.J. Saraiva, The inflammatory response to sciatic nerve injury in a familial amyloidotic polyneuropathy mouse model. Exp Neurol, 2014. 257: p. 76-87.

3. Sousa, M.M., et al., Familial amyloid polyneuropathy: receptor for advanced glycation end products-dependent triggering of neuronal inflammatory and apoptotic pathways. J Neurosci, 2001. 21(19): p. 7576-86.

#### P48 Real-life iluvien (fluocinolone acetonide) case sudy: rapid drying of the macula edema and improved vision within 1 year after therapy initiation IN ATTRV30M retinal angiopathy

##### João Beirão, Bernardete Pessoa, Maria-João Meneres, Pedro Meneres

###### Centro Hospitalar do Porto, Hospital Santo António, Porto, Portugal

####### **Correspondence:** João Beirão (brandaobeirao@gmail.com)

**Background**

A case showing sustained structural and functional responses 1 year after a single treatment with ILUVIEN (0.2 μg/day fluocinolone acetonide, FAc) despite suboptimal responses to anti-VEGFs’ treatment.

**Case report**

A liver transplanted ATTRV30M 48-year-old female patient with amyloid retinal angiopathy macular edema was first diagnosed in October 2015 and had a baseline visual acuity (VA) of 0.1 (Snellen chart) in the left eye. Central foveal thickness (CFT) was 502 microns. She was previous submitted to vitrectomy for vitreous amyloidosis and an Ahmed valvule implantation for glaucoma. The patient was treated with 5 intravitreal injections of bevacizumab and by May 2016, VA and CFT were largely unchanged (0.1 and 511 microns). An implant releasing FAc at a dosage of 0.2 μg/day (ILUVIEN) was administered in May 2016, and the optical coherence tomography indicated that the macula was dryer after 7 days (CFT was below 300 microns). This was sustained at 6 and 12 months after the treatment. VA improved to 0.4 within 14 days, and this was maintained after 12 months. Throughout the duration of this study, the intraocular pressure was 10 mmHg, and no glaucoma medication was administered.

**Conclusions**

In real-life practice, this ATTRV30M vitrectomized and Ahmed valvule implanted patient showed a suboptimal response to multiple intravitreal injections of bevacizumab. When subsequently treated with a single injection of ILUVIEN, there were large and rapid improvements in VA and CFT that were maintained for the following 1 year.

**Consent to publish**

Written informed consent was obtained from the patients involved in this study.

